# The evolutionary landscape of intergenic *trans*-splicing events in insects

**DOI:** 10.1038/ncomms9734

**Published:** 2015-11-02

**Authors:** Yimeng Kong, Hongxia Zhou, Yao Yu, Longxian Chen, Pei Hao, Xuan Li

**Affiliations:** 1 Key Laboratory of Synthetic Biology, Institute of Plant Physiology and Ecology, Shanghai Institutes for Biological Sciences, Chinese Academy of Sciences, Shanghai 200032, China; 2 Key Laboratory of Molecular Virology and Immunology, Institute Pasteur of Shanghai, Chinese Academy of Sciences, Shanghai 200031, China

## Abstract

To explore the landscape of intergenic *trans*-splicing events and characterize their functions and evolutionary dynamics, we conduct a mega-data study of a phylogeny containing eight species across five orders of class Insecta, a model system spanning 400 million years of evolution. A total of 1,627 *trans*-splicing events involving 2,199 genes are identified, accounting for 1.58% of the total genes. Homology analysis reveals that *mod(mdg4)*-like *trans*-splicing is the only conserved event that is consistently observed in multiple species across two orders, which represents a unique case of functional diversification involving *trans*-splicing. Thus, evolutionarily its potential for generating proteins with novel function is not broadly utilized by insects. Furthermore, 146 non-*mod trans*-spliced transcripts are found to resemble canonical genes from different species. *Trans*-splicing preserving the function of ‘breakup' genes may serve as a general mechanism for relaxing the constraints on gene structure, with profound implications for the evolution of genes and genomes.

## Abstract (web summary)

In intergenic *trans*-splicing, exons from two independent primary transcripts are joined. Here, the authors show that evolutionarily *trans*-splicing activity in insects is tightly controlled, with *mod(mdg4)* being a unique case of functional diversification involving trans-splicing.

## Graphical abstract

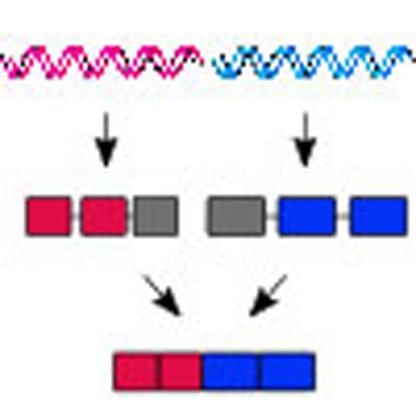

In eukaryotes, to form mature messenger RNA, the genetic code stored in non-contiguous units (exons) on nuclear DNA must be joined through a process called splicing. The splicing mechanism carries great potential for expanding the diversity and complexity of the eukaryotic transcriptome and proteome^1^,^2^. Compared with the commonly observed intra-molecular *cis*-splicing, intergenic *trans*-splicing, in which exons from two independent primary transcripts are joined, is less well studied. Its functional significance remains largely unknown, despite numerous cases documented in organisms from lower (such as, *Caenorhabditis elegans*^3^,^4^) to higher eukaryotes, including *Drosophila*^5^,^6^ and mammalian species^7^,^8^. In addition to the fact that many *trans*-splicing events in mammals are associated with malignant tissues, data from pilot ENCODE studies and other works have revealed the prevalence of chimeric RNAs in both normal tissues and transformed cell lines^9^. Other analyses also reported the involvement of an unexpected number of genes in ostensible *trans*-splicing events in mammalian cells^10^,^11^. One possible interpretation of these data is that these genes contributed to the existence of widespread *trans*-splicing activity in mammalian cells. Whether this phenomenon is functionally significant or represents background ‘splicing noise' has yet to be determined. Nonetheless, spliceosome-mediated pre-mRNA *trans*-splicing has been utilized to repair endogenous RNA species for the treatment of inherited and acquired diseases as a therapeutic application^12^.

Compared with trypanosomes and nematodes, in which frequent *trans*-splicing events involve a common short splicing leader^3^,^13^,^14^,^15^, intergenic *trans*-splicing events in insects are rare. The two best documented cases are *mod(mdg4)* (short for ‘modifier of *mdg4*') and *lola* (*longitudinals lacking*) in *Drosophila*^5^,^6^,^16^,^17^. *Mod(mdg4)* produces at least 31 splicing isoforms that share a common 5′-exon encoding the N-terminal BTB domain^17^,^18^. The 3′-variable region comes from independent primary transcripts, some located on the strand opposite the 5′-exons. The C2H2-type zinc-finger motif, called a FLYWCH domain, is found in most 3′-variable regions^18^. *Mod(mdg4)* has been implicated in position effect variegation, establishment of chromatin boundaries, nerve pathfinding, meiotic chromosome pairing and apoptosis^18^. *Lola* is an example of inter-allelic *trans*-splicing in *Drosophila*, as demonstrated by complementation tests and fly hybrid analysis using different allelic markers^19^. In addition, a recent study employing high-throughput transcriptome analysis identified additional inter-allelic *trans*-splicing events in flies, revealing more frequent inter-allelic events^16^.

The advance of high-throughput RNA-sequencing (RNA-seq) technology^2^,^20^,^21^,^22^ has enabled genome-wide analyses of *trans*-splicing events and has helped to reveal novel events in the silkworm *Bombyx mori*^23^ and in a variety of human cell types^9^,^24^. Furthermore, multi-lab collaborative projects such as FANTOM^25^ and modENCODE^26^, and public data cache such as NCBI SRA^27^ have generated and shared unprecedented resources, making large-scale evolutionary studies possible. To explore the landscape of *trans*-splicing events and to characterize their evolutionary dynamics and functions, we assemble a mega-data study of a phylogeny of eight species across five orders of class Insecta, representing a model system spanning 400 million years of evolution. Our goals are to address several essential questions concerning intergenic *trans*-splicing: (i) whether intergenic *trans*-splicing is functionally significant or merely represents splicing noise; (ii) to what extent intergenic *trans*-splicing events have impacted the transcriptome and proteome of an organism; (iii) how *trans*-splicing events originate and evolve; and (iv) whether and how *trans*-splicing is engaged in diversifying gene functions. With the availability of reference genome and transcriptome sequencing resources, we are able to perform a comprehensive screening of *trans*-splicing events across multiple insect orders and species. We have identified a total of 1,627 intergenic *trans*-splicing events, and many of those found in *Drosophila melanogaster* and *Danaus plexippus* are randomly selected for experimental validation. By systematically characterizing *trans*-splicing events in related insect species, we reveal the global profile and proteomic impact of these events, and also obtain crucial evidence about the origin and functions of intergenic *trans*-splicing events in insects. These findings deepen our understanding of the effect of *trans*-splicing on the molecular evolution of genes.

## Results

### Genome-wide identification of intergenic *trans*-splicing

**Figure 1:**
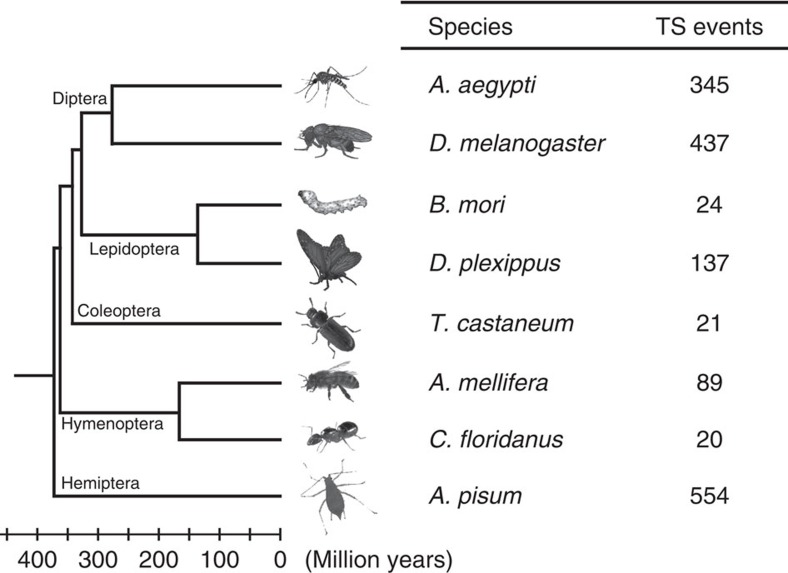
Identification of intergenic *trans*-splicing events in eight species across five orders of class Insecta. The branching order and divergence times are derived from the TimeTree database^62^. TS, intergenic *trans*-splicing events.

To characterize the landscape of intergenic *trans*-splicing events and to understand their evolution, we perform evolutionary analysis on well-defined insect lineages that offer some unique advantages for our study. Following careful research, we make use of a collection of eight representative species across five orders of class Insecta, including the Dipterans *D. melanogaster* and *Aedes aegypti*, the Lepidopterans *B. mori* and *D. plexippus*, the Coleopteran *Tribolium castaneum*, the Hymenopterans *Apis mellifera* and *Camponotus floridanus*, and the Hemipteran *Acyrthosiphon pisum* (see Methods for details and Fig. 1). In addition to their relationships within a clearly defined time-frame (refer to TIMETREE^28^ (Fig. 1)), a well-annotated genome and high-throughput RNA-seq data have been made available for each of these species. Thus, this phylogeny forms an ideal system for the systematic characterization of *trans*-splicing events. To identify candidate events, we first build an integrated pipeline modified from our previous work^23^ (see Methods for details). To eliminate false positives, we specify stringent criteria in our screening. For example, a *trans*-splicing event requires the support of both sequence reads covering the junction site and paired-end (PE) reads bridging exons of two different genes. A total of 1,627 *trans*-splicing candidate events are identified from the phylogeny, ranging from 20 in *C. floridanus* to 554 in *A. pisum* (Fig. 1 and Supplementary Data 1). Originally being parts of canonical gene models, the exonic elements involved in *trans*-splicing events present an intriguing question as to whether related splice sites are used simultaneously for both *cis*- and *trans*-splicing events. Notably, we find that elements in 1,268 out of 1,627 (77.93%) *trans*-splicing events are also involved in *cis*-splicing. In 859 events, both arms flanking junction sites are found in *cis*-spliced products at the same time, whereas in the other 409 events one of the arms is involved in *cis*-splicing (Supplementary Data 1).

To validate candidate *trans*-splicing events and estimate the success rate of our identification process, we scrutinize the subsets from *D. melanogaster, D. plexippus* and *B. mori.* First, we compare our identified events in *D. melanogaster* and *B. mori* with those confirmed by previous studies^5^,^18^,^23^. All nine *mod(mdg4) trans*-spliced isoforms joining exons located on opposite strands in *D. melanogaster*^5^,^18^ are identified by our pipeline (Supplementary Table 1). However, the 22 *mod(mdg4)* isoforms joining exons on the same strand are correctly not included in our set, as overlapping or neighbouring genes on the same strands are excluded by our filter. In *B. mori*, 7 of 9 *mod(mdg4)* and 9 of 12 *non-mod trans*-spliced products identified by Shao *et al*.^23^ are also found in our set. We look further into the two *mod* and three non-*mod* events previously reported for *B. mori* that have been missed by our pipeline. They are found to be supported by fewer than two chimeric reads and thus are not called in our search because of the more stringent thresholds instituted by our study compared with earlier work. Second, we take an experimental approach to examine our *trans*-splicing events in the fly *D. melanogaster* and the butterfly *D. plexippus* by reverse transcription–PCR (RT–PCR) and Sanger sequencing. It has been reported previously that template switching in reverse transcription can produce artefacts mistaken for *trans*-spliced sequences, which are mostly non-canonical and reverse transcriptase (RTase) dependent^29^. To mitigate the issue caused by template switching, we employ RT–PCR using Moloney Murine Leukemia Virus (MMLV)-derived RTase and Avian Myeloblastosis Virus (AMV)-derived RTase in parallel experiments. Experiments with different RTases were shown to be an effective diagnostic tool for template switching artefacts, and the two-RTase routine was found to produce results consistent with the RNase protection assay^24^. We first calibrate our protocol using the six *D. melanogaster mod(mdg4) trans*-spliced isoforms as positive controls (see Methods for details). Our method is able to amplify and sequence-verify all six isoforms from mixed fly samples (Supplementary Fig. 1). Then, we randomly pick 17 additional *trans*-splicing events from our *D. melanogaster* subset for experimental validation, 11 (64.7%) of which are found to produce the predicted PCR-amplification products verified by Sanger sequencing (Supplementary Fig. 1; Supplementary Tables 2 and 3). In *D. plexippus*, 14 of 18 (77.8%) predicted *mod(mdg4) trans*-splicing isoforms, and 10 of 10 (100%) non-*mod trans*-splicing products are validated from *D. plexippus* mixed tissue samples (Supplementary Table 2 and Supplementary Fig. 1). Notably, our validation rates are in line with those for *B. mori*^23^, and all experimentally validated *D. melanogaster* and *D. plexippus trans*-splicing events are found to ligate at the precise splicing-junction sites that are confirmed by Sanger sequencing, demonstrating the efficiency and consistency of our approach and strengthening confidence in our analysis pipeline.

### Global profile of intergenic *trans*-splicing events

**Figure 2:**
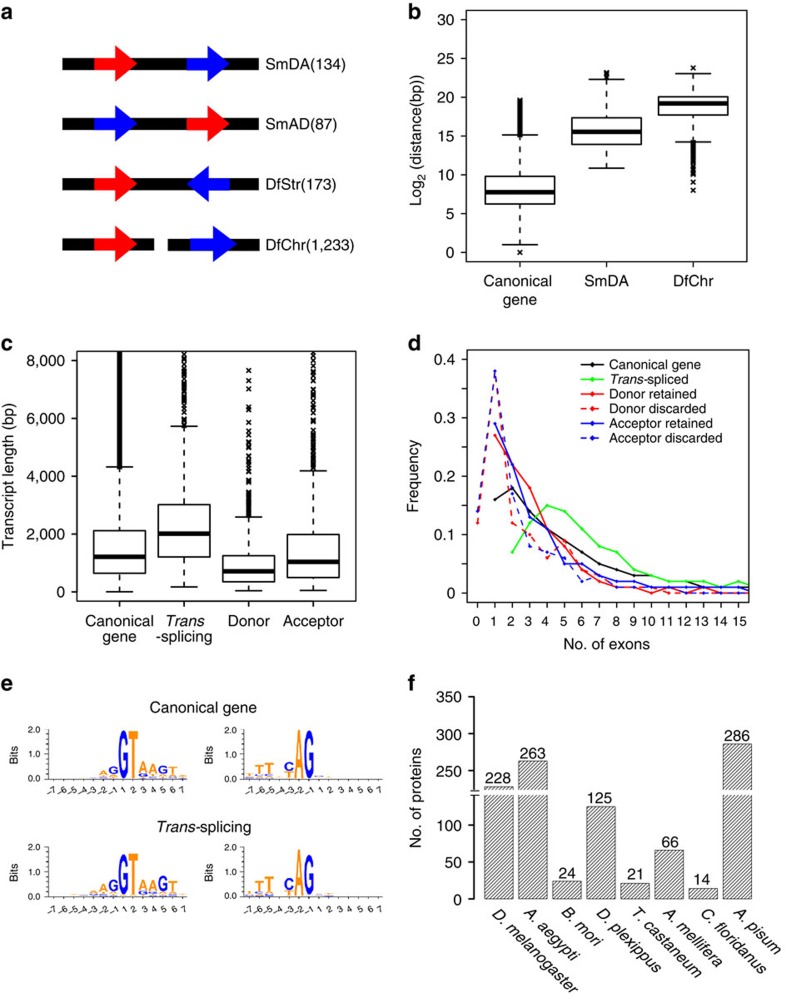
Characterization of intergenic *trans*-splicing events in insects. (**a**) Schematic diagram of four organizational types of donor and acceptor genes involved in intergenic *trans*-splicing. The arrows denote the direction of transcripts and their relative positioning on the chromosome; donor genes are in red and acceptor genes are in blue. The counts for each type are indicated in parentheses to the right. DfChr, different chromosomes/scaffolds; DfStr, different strand; SmAD, same chromosome with acceptor upstream of donor; SmDA, same chromosome with donor upstream to acceptor. (**b**) Distances between canonical exons and between donor and acceptor genes of *trans*-splicing shown in box plot. The bottom and top of the box represent the first and third quartiles, and the band inside the box represents the second quartile (the median). The whiskers extend 1.5 interquartile range (third quartile minus first quartile) upward from the third quartile, and downward from the first quartile. Outliers are marked with cross. The putative distance for DfChr is computed only for those sites on different scaffolds of unknown chromosomal location, assuming that donor and acceptor scaffolds are located on the same chromosome and that both genes are arranged in the same orientation, with the donor upstream of the acceptor. (**c**) Length of canonical transcripts, *trans*-spliced products and donor and acceptor segments of *trans*-splicing events shown in box plot. The parameters used in the box plots are the same as those of **b**. (**d**) Frequency distribution of transcripts with different number of exons. The *x* axis indicates the number of exons for each transcript types. Donor retained, *trans*-spliced transcript with retained donor segments; donor discarded, transcript with discarded donor segments; acceptor retained, *trans*-spliced transcript with retained acceptor segments; and acceptor discarded, transcript with discarded acceptor segments. (**e**) Nucleotide conservation at the junction sites of canonical and *trans*-spliced genes, shown with WebLogo^63^(v3.3). The *y* axis indicates the relative frequency of each nucleic acid in bits (a unit of entropy). (**f**) Predicted proteins from intergenic *trans*-splicing events in insects. To qualify, translation must initiate within the donor segment and must contain at least ten amino acids in both the donor and acceptor segments.

The 1,627 identified *trans*-splicing events involve 2,199 of 139,446 total genes from the eight insect species. The fraction (∼1.58%) of genes involved indicates a low *trans*-splicing activity. To characterize the *trans*-splicing events and their distribution pattern, the identified events are analysed using their respective reference genomes. In a *trans*-splicing event, the donor gene and the acceptor gene are defined as the gene that ‘donates' the upstream exon and the gene that provides the downstream exon to be joined to (‘accept') the donor, respectively. Based on the chromosomal or scaffold locations and transcript orientations of the donor and acceptor genes, the 1,627 events are grouped into four types (Fig. 2a). To detect possible mix-up between canonical *cis*-splicing genes and types SmDA and DfChr, the distances between exons in *cis*-splicing are compared with the distances between donors and acceptors in types SmDA and DfChr. Note the distances between donors and acceptors in type DfChr are computed for those on different scaffolds of unknown chromosome location (refer to Fig. 2a legend). The median distance between donor and acceptor genes (∼46.4 kb for SmDA and ∼606 kb for DfChr) is significantly greater than that between canonical exons (∼0.21 kb; Fig. 2b and Supplementary Fig. 2). This result indicates that the likelihood of false positives due to incomplete genome assembly data is very low. Notably, the greater distance bridged by the *trans*-splicing mechanism, as quantified by our statistics, represents an intriguing phenomenon in RNA processing.

The sizes of the *trans*-spliced transcripts are analysed and compared with those of *cis*-spliced transcripts. The median size of the *trans*-splicing products is significantly larger, mostly due to the size of the 3′-acceptor (Fig. 2c). These attributes of the *trans*-spliced transcripts are consistent among the eight investigated species (Supplementary Fig. 3). Upon comparing the exons of the *cis*-spliced and *trans*-spliced products, we find a higher number of exons in the *trans*-spliced products than in *cis*-spliced (Fig. 2d). Moreover, the last exon of the donor and the first exon of the acceptor are the least retained in *trans*-spliced products. The nucleotide composition surrounding the *trans*-splicing sites is summarized in Fig. 2e, with a conserved pattern with a signature GU-AG consensus^30^ similar to that in *cis*-splicing. The common pattern supports that both *trans*- and *cis*-splicing utilize the same splicing machinery^12^,^31^.

To evaluate the impact on the proteome of the formation of chimeric protein species through *trans*-splicing events, we analyse the proteins translated from the *trans*-spliced transcripts. Overall, 63% (1,027/1,627) of the *trans*-splicing events contribute to the formation of new chimeric proteins (Fig. 2f and Supplementary Data 2). Assuming a single protein for each canonical gene, these 1,027 new chimeric proteins amount to ∼0.73% of all encoded protein species. Notably, 67.3% (691/1,027) of the chimeric proteins are translated in the same frame as both the donor and acceptor genes. The bias towards joining two existing CDSs is significant (*χ*^2^ test: *P*-value <2.2E-16). This global profile of intergenic *trans*-splicing events, which to our knowledge is the first completed across multiple orders of insects, illustrates both similarities to and differences from *cis*-splicing.

### Conserved *trans*-splicing events are rare except *mod(mdg4)*

To understand how the *trans*-splicing events are related and how they evolved, we build a homology matrix via pairwise comparison using BLASTP among the 1,027 *trans*-spliced coding transcripts (see Methods for details). By definition, a conserved *trans*-splicing event requires both peptides encoded by its donor and acceptor segment to match those of events from different species. Our stringent criteria exclude cases of partial matching by either the donor or the acceptor segment alone. The matrix is then screened for conserved events, and surprisingly, with the exception of *mod(mdg4)*, no other events are found to meet our criteria (Supplementary Table 4). For the 23 conserved *mod(mdg4)*-like events, 9 were previously reported for *B. mori* and *D. melanogaster* (Supplementary Table 1), whereas the remaining 14 are new *mod(mdg4)*-like events discovered in *A. aegypti* and *D. plexippus*.

### *Mod*-like *trans*-splicing conserved in Lepidoptera and Diptera

Our multi-genome screening result indicates that *mod(mdg4)*-like events represent a unique case of intergenic *trans*-splicing conserved in insects. Therefore, it is imperative that we characterize their activity more broadly for class Insecta to address the question of how *mod(mdg4)*-like *trans*-splicing events originated and evolved. To this end, we add two more species, *Plutella xylostella* and *Anopheles gambiae*, to our analysis.

**Figure 3:**
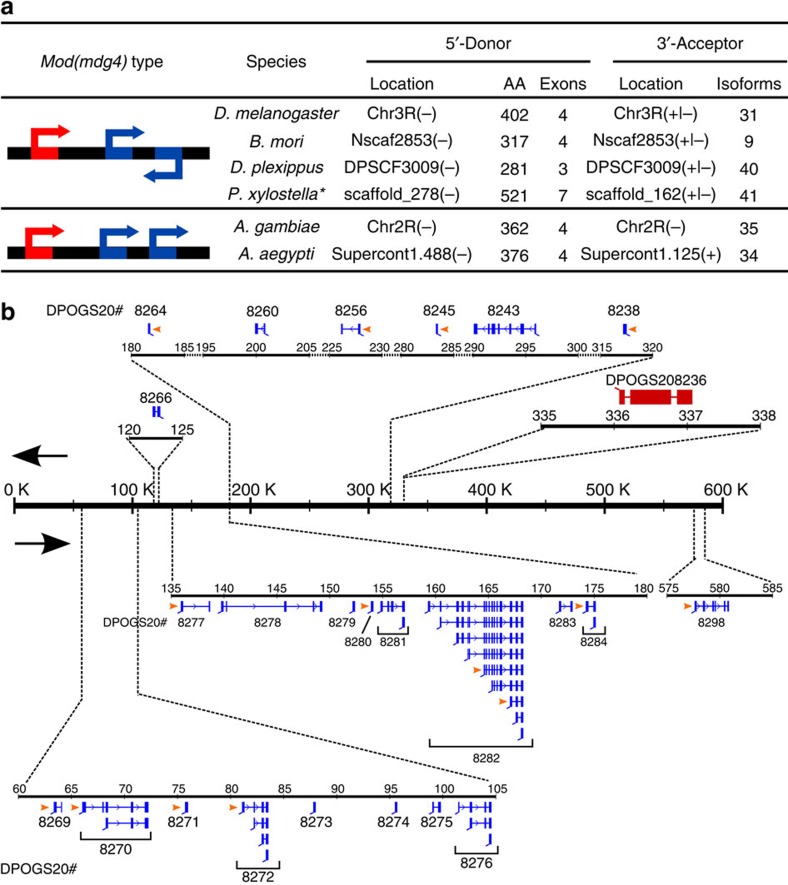
*Mod(mdg4)*-like *trans*-splicing events conserved in insects. (**a**) Two different types of *mod(mdg4)*-like *trans*-splicing. AA, number of amino acids; Red bar, donor transcript (5′-common exons); blue bar, acceptor transcript (3′-alternative exons). Arrows denote direction of transcripts. *Assuming that scaffold_278 and scaffold_162 are located on the same chromosome in *P. xylostella*. (**b**) Diagram of the *mod(mdg4)* locus in *D. plexippus*. Red bar, donor transcript (5′-common exons); blue bar, acceptor transcript (3′-alternative exons). Black arrows indicate strand direction. *Mod(mdg4)* isoforms validated by RT–PCR are marked by orange arrows. The RT–PCR is performed as described in Methods using mixed *D. plexippus* samples (*n*=3). The name of each acceptor is denoted by DPOGS20 (prefix) plus the four-digit label of blue bar(s).

*Mod(mdg4) trans*-splicing was first reported in *Drosophila*^5^,^6^,^17^,^18^, followed by *A. gambiae*^17^ and *B. mori*^23^. In the current work, new cases of *mod(mdg4) trans*-splicing are identified in *D. plexippus, P. xylostella* and *A. aegypti* (Fig. 3 and Supplementary Table 5)*. mod(mdg4)*-like transcripts represent an ancient gene traced to the ancestor of the insect lineage. Canonical *cis*-splicing genes homologous to *mod(mdg4)* transcripts are found in the Coleopteran *T. castaneum*, two Hymenopterans *A. mellifera* and *C. floridanus*, and in the distant relative Branchiopodan *Daphnia pulex*^32^ (see Methods for details; and Supplementary Fig. 4). *Mod(mdg4) trans*-splicing products are formed by joining 5′-donor exons with one or a combination of 3′-acceptor exons. The 5′-donor exons are noticeably variable between species, comprised of between 3 and 7 exons (Fig. 3a and Supplementary Table 5). Strikingly, the 3′-acceptor has a large number of variable exon combinations: 31, 9, 40, 41, 35 and 34 different combinations are observed, respectively, in *D. melanogaster, B. mori*, *D. plexippus, P. xylostella, A. gambiae* and *A. aegypti* (Fig. 3a).

The structure of *mod(mdg4)*-like gene loci can be divided into two groups based on configuration. In *D. melanogaster*, *B. mori*, *D. plexippus* and possibly *P. xylostella*, the donor and acceptor genes are on the same chromosome, but the acceptors' exons could be found on both DNA strands, either on the same strand or opposite to the donor exon (Fig. 3a). In contrast, *A. gambiae* exhibits cases in which both the donor and acceptor exon of *mod(mdg4)* were located on the same DNA strand^17^. The *A. gambiae mod(mdg4)* gene was likely to undergo *trans*-splicing because of the similar donor–acceptor proximity and exon–intron structure between the *A. gambiae* and *D. melanogaster mod(mdg4)* loci^5^,^17^. *A. aegypti*, another mosquito species, may have the same configuration, assuming that its *mod(mdg4)* donor and acceptors are located on the same chromosome (Fig. 3a). The distances between the donor and the acceptors range from 950 kb to 1.280 Mb. The observation of two distinct patterns at *mod(mdg4)* loci indicates that a structural rearrangement occurred within the Dipteran lineage after the divergence of flies and mosquitoes.

*Mod(mdg4)*-like *trans*-splicing in *D. plexippus* is an intriguing and novel case. It presents at least 40 different isoforms stemming from alternative exon combinations from 24 acceptor genes located on scaffold DPSCF300079 distributed over a locus of 550 kb (Fig. 3b). In total, 7 and 33 isoforms took the acceptor exons from the same strand or opposite strand to the donor exons, respectively. However, notably different from the other species, *D. plexippus* has a special acceptor gene, DPOGS208298, located on the opposite strand upstream of the donor gene (Fig. 3b; marked as 8298). Furthermore, frequent alternative *trans*-spliced *mod(mdg4)* transcripts are observed in *D. plexippus*. For example, acceptor gene DPOGS208282 has nine alternative isoforms with variable combinations of exons (Fig. 3b and Supplementary Table 5). To verify the presence of alternative *trans*-spliced transcripts, we have performed RT–PCR/sequencing analysis with specific primers designed for 18 of the *mod(mdg4)* isoforms. Fourteen are validated by PCR-amplification products of the predicted size (Fig. 3b, marked with orange arrows; Supplementary Fig. 1). It is worth mentioning that the *trans*-splice sites and the surrounding sequences in *mod(mdg4)* are not conserved between insect species, because they are located outside the conserved BTB and FLYWCH domains.

### Diversification of *mod(mdg4)* via alternative *trans*-splicing

**Figure 4:**
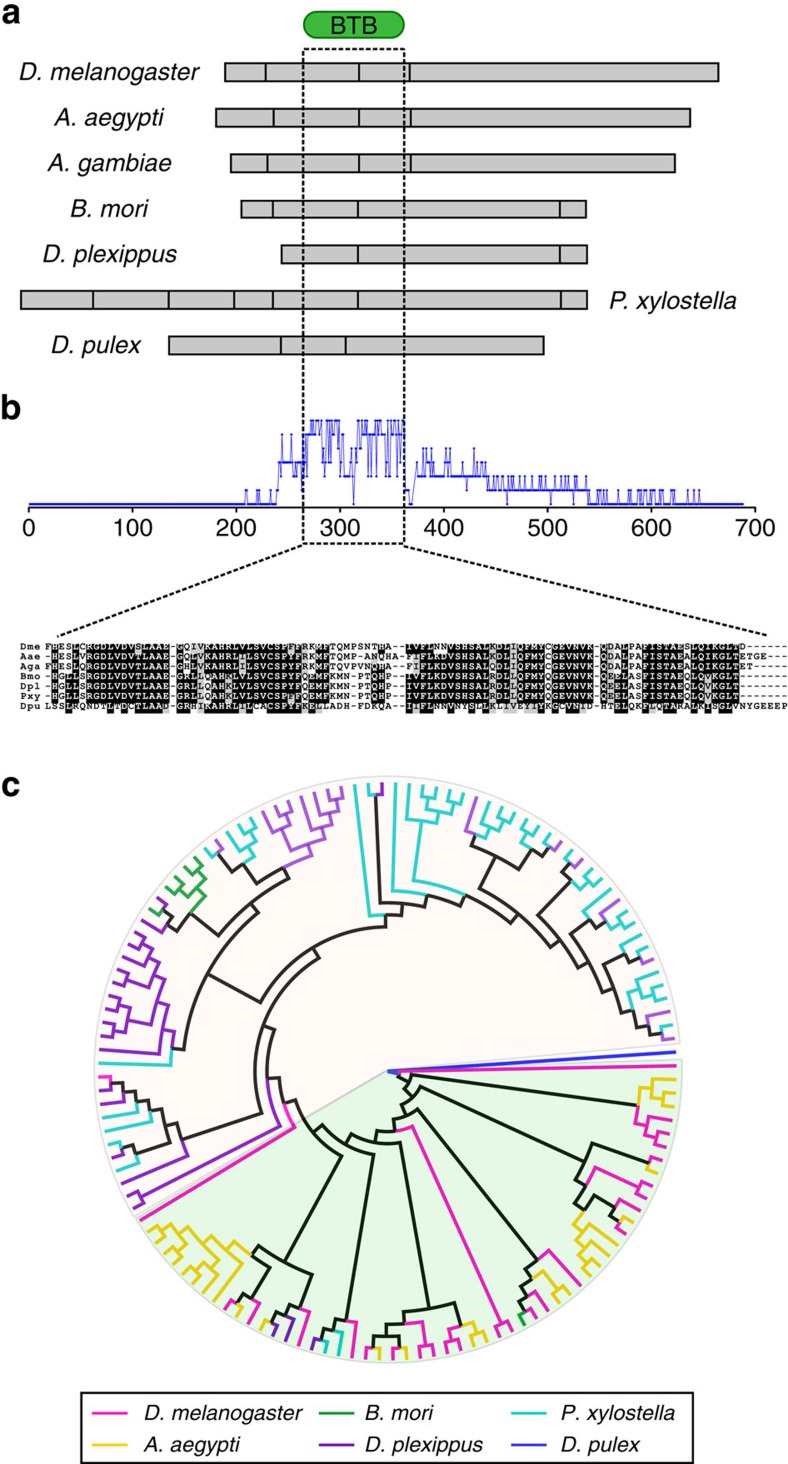
Sequence diversification of *mod*(*mdg4*) products throughout evolution. (**a**) Exon structure of the *mod(mdg4)* N-terminus. The BTB domain-encoding region is marked by a dashed box. Grey box, exon. (**b**) Sequence similarity of N-terminal regions. Number of conserved amino acid at each position is indicated by blue curve. Sequences are aligned based on the BTB domain model using HMMalign^59^ (see Methods for details). Only alignment of the conserved region is shown. Dme, *D. melanogaster*; Aae, *A. aegypti*; Aga, *A. gambiae*; Bmo, *B. mori*; Dpl, *D. plexippus*; Pxy, *P. xylostella*; Dpu, *D. pulex*. (**c**) Phylogenetic analysis of C-terminal FLYWCH domains using Maximum Likelihood analysis (see Methods for details). FLYWCH domains from different species are marked in distinct colours. The Dipteran and Lepidopteran groups are separated by different background colours. The same tree with detailed labelling is provided in Supplementary Fig. 5.

*Daphnia* has a canonical *cis*-spliced *mod(mdg4)*-like homologue (Supplementary Fig. 4). The same is true for species of the Coleopteran and Hymenopteran lineages, including *T. castaneum, A. mellifera* and *C. floridanus* (Supplementary Fig. 4). To understand the evolutionary trajectory of *mod(mdg4)*, we analyse the donor and acceptor genes that code protein sequences. Although the number of donor exons varies, the N-terminal segment of the *mod(mdg4)* protein contains a highly conserved BTB domain (∼110 amino acids; see Methods for details and Fig. 4a), in which the homology between the N-terminus of *mod(mdg4)* proteins from different species is primarily found (Fig. 4b), implying a conserved function and critical role for the BTB domain. It is worth noting that the BTB domain has a zinc finger structure that can mediate protein–protein or protein–DNA interactions^33^.

The C-terminus of the *mod(mdg4)* protein is highly variable in size, depending on the combination of exons retained from acceptor genes. The FLYWCH domain (∼60 amino acids) is found in most acceptor isoforms (for example, 26 of 31 in *D. melanogaster*, 6 of 9 in *B. mori*, 25 of 34 in *A. aegypti*, 37 of 41 in *P. xylostella* and 37 of 40 in *D. plexippus*). To reveal its evolutionary path, consensus regions of the aligned FLYWCH domains are used to construct a phylogenetic tree using the Maximum Likelihood (ML) approach, in which the *D. pulex* domain is used as the outgroup (see Methods for details; Fig. 4c and Supplementary Fig. 5). The result of this analysis places the FLYWCH domains into two distinct groups between Dipterans and Lepidopterans, in which clusters containing FLYWCH domains from closely related species have formed. The duplications of the FLYWCH domain occurred separately within the Dipteran and Lepidopteran lineages after their divergence, because few interwoven FLYWCH domains are present across the two orders. Then, along the way several rounds of duplication took place in the lineages leading to *D. melanogaster*, *B. mori*, *A. aegypti*, *D. plexippus* or *P. xylostella.* Consistent with this hypothesis, FLYWCH domains clustered in one branch often correspond to tandem repeats of exons at the same acceptor gene locus (Fig. 4c).

### Non-*mod trans*-spliced products resemble regular transcripts

Because non-*mod* events represent the majority of *trans*-splicing events identified, it is of particular interest to determine how these events originated and evolved. Thus, we perform a pairwise comparison via BLASTP between the non-*mod trans*-spliced coding transcripts and canonical genes from the eight species (see Methods for details). Although the non-*mod trans*-spliced products do not match among themselves, a striking number have homologues among canonical genes in different species (Table 1). Under stringent thresholds of 50% identity and 90% coverage between matching transcripts, 146 non-*mod trans*-spliced transcripts find at least one match in different species. In other words, these non-*mod trans*-spliced transcripts resemble canonical genes from other species. This intriguing finding suggests that a significant fraction of *trans*-splicing events are likely the result of regular *cis*-splicing gene ‘break-up' (Supplementary Data 3). A similar phenomenon was observed in *C. elegans*, in which the *trans*-spliced product of *eri-6* and *eri-7* was identical to the single contiguous gene, CBG03999, in *Caenorhabditis briggsae*^4^.

**Figure 5:**
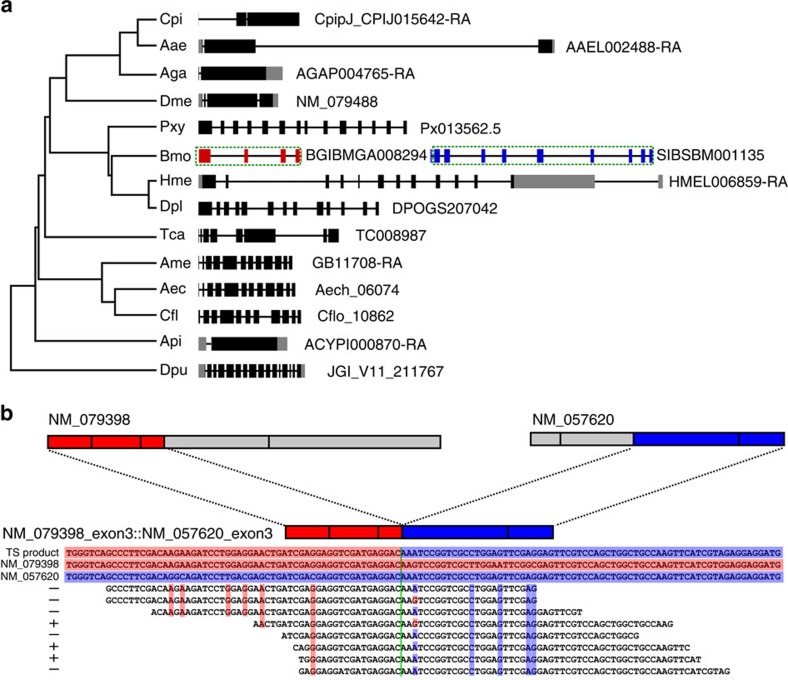
Examples of non-*mod trans*-splicing events. (**a**) Comparison of BGIBMGA008294_exon4::SIBSBM001135_exon2 *trans*-splicing in silkworm and its homologues from other species. Exons retained in the *trans*-spliced product are enclosed in dashed boxes. Black block, coding exons; grey block, untranslated exons; red block, donor exons; blue block, acceptor exons. Cpi, *C. pipiens*; Aae, *A. aegypti*; Aga, *A. gambiae*; Dme, *D. melanogaster*; Pxy, *P. xylostella*; Bmo, *B. mori*; Hme, *H. melpomene*; Dpl, *D. plexippus*; Tca, *T. castaneum*; Ame, *A. mellifera*; Aec, *A. echinatior*; Cfl, *C. floridanus*; Api, *A. pisum*; Dpu, *D. pulex*. (**b**) Schematic diagram of *trans*-splicing between two paralogues, NM_079398 and NM_057620. Red denotes donor exons, and blue indicates acceptor exons. Sequence reads covering the junction site are aligned, with critical residues highlighted in background colours consistent with those of the donor or acceptor.

The relationship between a *trans*-spliced transcript and its canonical gene homologues in other species is exemplified by the *trans*-splicing observed between BGIBMGA008294_exon4 and SIBSBM001135_exon2 in *B.mori*, which was validated experimentally by allele-specific RT–PCR^23^. Besides the *trans*-spliced form, no canonical transcript involving either the donor or acceptor is found in *B.mori* using RNA-seq data. However, its homologous *cis*-spliced canonical gene is found in daphnia (JGI_V11_211767) and 12 other insects, including *Culex pipiens*, *A. aegypti*, *A. gambiae*, *D. melanogaster*, *P. xylostella*, *Heliconius melpomene*, *D. plexippus*, *T. castaneum*, *A. mellifera*, *Acromyrmex echinatior*, *C. floridanus* and *A. pisum* (Fig. 5a and Supplementary Data 3). The BGIBMGA008294_exon4::SIBSBM001135_exon2 transcript in *B. mori* bears an exon–intron structure similar to that of other Lepidopterans, including *P. xylostella* (14 exons), *H. melpomene* (13 exons) and *D. plexippus* (13 exons). These *trans*-splice sites are also conserved in the most closely related species. However, gene structure became dramatically different as the phylogenetic distance increased between species. Homologues of the Hemipteran *A. pisum* and the Dipterans *C. pipiens*, *A. aegypti*, *A. gambiae* and *D. melonagaster* have exon counts of 2, 3, 3, 2 and 4, respectively, indicating that an exon-fusion process took place within the Hemipteran and Dipteran lineages. Given that the outgroup daphnia homologue, JGI_V11_211767, has 16 exons, this family of genes is likely to have undergone multiple rounds of exon breakup and fusion during the process of lineage expansion. Thus, we conclude that *trans*-splicing between BGIBMGA008294_exon4 and SIBSBM001135_exon2 originated in the *B. mori* lineage, where the breakup of the canonical homologue took place. Our analysis of the structural history of *trans*-spliced transcripts and their canonical gene homologues reveals a picture of frequent structural shuffling in parallel with the expansion of the insect lineage. Based on this observation, we propose that intergenic *trans*-splicing serves to preserve the function of broken genes following genomic mishaps. *Trans*-splicing allows for the breakup of genes without a complete loss of function, which increases the tolerance to genome rearrangement and structural changes during evolution. This may be a general mechanism to relax constraints on gene structure and thus would exert a profound effect on the evolution of genes and genomes. Such a function may bear special significance in the case of rapidly expanding organisms such as insects, whose genomes continue to evolve rapidly.

Although our pipeline cannot differentiate *trans*-splicing events between alleles of the same gene (that is, inter-allelic), which requires preknowledge of distinguishable allelic features, we reason that between-paralogue *trans*-splicing events would resemble those attributed to inter-allelic *trans*-splicing. By screening pairs of donor and acceptor genes in *trans*-spliced coding transcripts from the same species, we find that fewer than 5.8% of cases (60/1,027) possess homology between donor and acceptor sequences (Supplementary Table 6). Thus, *trans*-splicing events between likely paralogues account for a small fraction of the total events identified via our pipeline. We select some of these cases for visual evaluation. For example, the *D. melanogaster* calcium ion-binding protein genes *TpnC73F* and *TpnC47D* are paralogues located on chromosomes 3L and 2R, respectively. Our analysis finds that the *TpnC73F* transcript, NM_079398, donates the first three exons to the last two exons of the *TpnC47D* transcript, NM_057620, thereby forming a five-exon *trans*-spliced product (Fig. 5b) coexisting with their *cis*-spliced forms. RNA-seq reads covering the junction site reveal the precise joining of two exons with source-specific single-nucleotide variants aligned on both arms. The data of between-paralogue *trans*-splicing events support the inter-allelic results previously reported for *Drosophila*^16^,^19^, suggesting that both between-paralogue and inter-allelic *trans*-splicing may take place via a similar mechanism. We further speculate that between-paralogue and inter-allelic *trans*-splicing events may play a role in creating a new mix of functional products.

## Discussion

To address the question of how intergenic *trans*-splicing events originate and evolve and to what extent they impact the proteome, we assemble a mega data analysis on a phylogeny of eight species spanning multiple orders in class Insecta, significantly expanding the scope of *trans*-splicing study. By design, this model system represents the most explosive expansion of living organisms on the Earth, spanning an evolutionary timeframe of ∼400 million years. Interrogation of this model system provides us a historic view of the landscape of intergenic *trans*-splicing events and deepens our understanding of the function and evolutionary dynamics of *trans*-splicing.

A total of 1,627 *trans*-splicing events involving 2,199 genes are identified. Consistent with previous results^9^,^11^ from the ENCODE project and other studies, we observe the involvement of a small fraction (1.58%) of total genes, accounting for 0.73% of encoded protein species. The low occurrence of ‘background' events does not support the existence of widespread *trans*-splicing activity in insects, in agreement with an early study performed in *Drosophila*^16^. However, our evidence also argues against the hypothesis that intergenic *trans*-splicing events are merely ‘splicing noise.' *Mod(mdg4)*-like *trans*-splicing events are found in multiple species across Diptera and Lepidoptera, which share a common ancestor from which they diverged ∼360 million years ago. It is likely that the elements for *trans*-spliced *mod(mdg4)* originated from a canonical gene in their common ancestor and were separated into donor and acceptor genes during the expansion and divergence of the insect lineages. The start of *mod(mdg4)*-like *trans*-splicing was followed by the duplication and expansion of the acceptor genes encoding the FLYWCH domain. The expansion of acceptor genes enlarged the repertoire of *mod(mdg4)* isoforms that might be used for diversified functions, which in turn enabled their complex regulation during the development and adaptation of these insects. Notably, this unique case of generating functional diversity through intergenic *trans*-splicing might not be achieved through alternative *cis*-splicing because of limitations imposed on canonical *cis*-splicing genes. Remarkably, *mod(mdg4)*-like *trans*-splicing remains the only event that is conserved and engaged in the diversification of gene function in insect lineages, despite the fact that the *trans*-splicing phenomenon has been observed in organisms as early as the primitive metazoans (for example, the nematode *C. elegans*)^3^,^4^. Taken together, our data and other studies indicate that intergenic *trans*-splicing appears to be tightly controlled through evolution. Its potential to create new functions by combining remote coding exons is not broadly utilized by insects throughout their evolutionary history.

The finding that some non-*mod trans*-splicing transcripts resembling canonical *cis*-splicing genes from different species is somewhat unexpected. Albeit relatively small in number, these transcripts present strong evidence that *trans*-splicing acts to preserve occasionally broken genes during the structural shuffling of the genome and during exon-breakup-fusion events. In this scenario, *trans*-splicing mechanism in insects acts more as a ‘saviour' than ‘creator' of gene function. *Trans*-splicing appears to increase the tolerance to genome structural changes during evolution by relaxing constraints on gene structure, which in turn has profound implications for the evolution of genes and genomes.

## Methods

### Reference genome and collection of RNA-seq data

Insect genome reference sequences are retrieved from various public databases. They include R5/dm3 from UCSC^34^ for *D. melanogaster*; AaegL1 from VectorBase^35^ for *A. aegypti*; silkworm_genome_v2.0 from SilkDB^36^ for *B. mori*; Dp_genome_v3 from MonarchBase^37^ for *D. plexippus*; Tcas 3.0 from BeetleBase^38^ for *T. castaneum*; Amel_2.0 from BeeBase^39^ for *A. mellifera*; Cflo_v3.3 from Fourmidable^40^ for *C. floridanus*; assembly2_scaffolds (version 2) from AphidBase^41^ for *A. pisum* and scaffold sequences of version 2 from DBM-DB^42^ for *P. xylostella*.

For each reference version, the most recent gene set is retrieved. These include R5/dm3 for *D. melanogaster*; AaegL1.4 for *A. aegypti*; annotation modified by Shao *et al*.^23^ for *B. mori*; OGS v2.0 for *D. plexippus*; Tcas 3.0 for *T. castaneum*; Amel_1.1 for *A. mellifera*; Cflo_v3.3 for *C. floridanus*; ACYPI v2.1b for *A. pisum* and OGSv1.0 for *P. xylostella*. In addition, for the evolutionary analysis of *trans*-spliced coding transcripts, annotated gene sequences with exon–intron structure are retrieved from public databases: AgamP3.7 from Vectorbase^35^ for *A. gambiae*; gene set of *C. pipiens* assembly 3 from *Culex pipiens* Database^43^ for *C. pipiens*; gene set v1.1 from Butterfly Genome Database^44^ for *H. melpomene*; gene set v3.7 from Fourmidable^40^ for *A. echinatior* and JGI release v1beta from wFleaBase^45^ for *D. pulex*.

High-throughput RNA-seq data are obtained from the NCBI Sequence Read Archive^46^ (SRA; http://www.ncbi.nlm.nih.gov/sra), except where specifically indicated otherwise. The RNA-seq data for *D. melanogaster* are obtained from 30 developmental stages of the embryo, pupae, larvae, adult male and adult female^47^. Their accession codes are: SRX007811, SRX008008, SRX008012, SRX008016, SRX008020, SRX008024, SRX008028, SRX008157, SRX008005, SRX008009, SRX008013, SRX008017, SRX008021, SRX008025, SRX008029, SRX010758, SRX008006, SRX008010, SRX008014, SRX008018, SRX008022, SRX008026, SRX008155, SRX012269, SRX008007, SRX008011, SRX008015, SRX008019, SRX008023, SRX008027, SRX008156, SRX012270 and SRX012271. The RNA-seq data for *A. aegypti* are obtained from four developmental samples, including male testes, male carcass, female ovary and female carcass^48^. Their accession codes are: SRX316667, SRX316704, SRX316706 and SRX316705. The RNA-seq data for *B. mori* are obtained from 77 mixed samples of various developmental stages^23^. The accession code is SRX084698. The RNA-seq data for *D. plexippus* are obtained from mixed samples of all developmental stages^49^. Its accession code is SRX191135. The RNA-seq data for *P. xylostella* are obtained from four developmental stages of egg, larva, pupa and adult^50^. Their accession codes are SRX056231, SRX056232, SRX056233 and SRX056234. The RNA-seq data for *T. castaneum* are obtained from whole larvae^51^. Its accession code is ERP001667. The RNA-seq data for *A. mellifera* are obtained using bees from colonies of single drone inseminated queens^52^. Their accession codes are SRR498622, SRR499808, SRR499882, SRR499883, SRR499919, SRR499920, SRR499992, SRR499993 and SRR499995. The RNA-seq data for *C. floridanus* are obtained from whole body samples of a queen and a virgin queen^53^. The accession codes are SRX091808 and SRX091809. The RNA-seq data for *A. pisum* are obtained from two samples taken during the late steps of sexual embryogenesis. The accession codes are SRX040564 and SRX040565 (ref. 54).

### Bioinformatics methods for identifying *trans*-splicing events

Low-quality reads are filtered using a previously described protocol^23^ with the following modifications. First, reads are only retained if at least 60% of the bases have a quality score ⩾20. Second, both the 5′ and 3′ ends of the reads are trimmed to remove bases with a low-quality score (<20). Third, trimmed reads with more than one ‘N' are discarded. The quality of each insect genome is assessed by mapping single-end and PE RNA-seq reads separately to the genome assemblies for the respective species (Supplementary Fig. 7).

The pipeline for the identification of *trans*-splicing events is modified from an earlier study^23^ (Supplementary Fig. 6). Briefly, quality-filtered reads are aligned to a reference genome by TopHat^55^ (v2.0.9) with the default parameters. Mapped reads are filtered, and unmapped reads are processed by Bowtie^56^ (v1.0.0) for another round of filtering with the default parameters. These two filtering steps ensure the removal of reads derived from canonical *cis*-splicing. The retained reads are then mapped to a between-gene exon–exon fusion library using the default parameters of Bowtie^56^ (v1.0.0). A library is constructed for each of the eight insect species based on their annotated gene models and defined exon–intron boundaries. Each library include all possible fusion sequences between exons of two different genes within a single species. A *trans*-splicing event is identified only when a match is found to meet the following criteria: (i) the junction site is covered by at least two non-redundant reads, one of which must be perfect match; (ii) reads covering the junction site must match to both arms for at least 20 bp each; (iii) in addition to covering the junction site, the *trans*-splicing event must be supported by at least one of the PE reads bridging the two flanking regions; (iv) *trans*-splicing candidate events between overlapping or neighbouring genes are further examined, and events between genes with the same orientation are removed to eliminate false positives stemming from conjoined genes resulting from possible transcriptional readthrough. Using this approach, with the above filters designed to eliminate the false positives, the success rate of the identified events, validated by the RT–PCR assay as described below, is found to be independent of the quality of the genome assembly.

### Experimental validation of *trans*-spliced transcripts

Wild-type (Canton-S) *D. melanogaster* samples are collected from four developmental stages, including embryo (24 h after egg laying), third-instar larvae, pupae (third day after pupating) and adult (2–3 days after eclosion). Adult *D. plexippus* samples are provided by Dr Steven Reppert (University of Massachusetts) and are stored in RNAlater (Life Technologies) during shipment. Total RNA is isolated separately from each *D. melanogaster* sample, and from the *D. plexippus* thorax and head, using an RNeasy Plus Mini Kit (Qiagen) with gDNA Eliminator Spin Columns to remove gDNA contamination.

RNA is reverse-transcribed using a RevertAid First Strand cDNA Synthesis Kit (Thermo Scientific) with oligodT and random primers. To detect template switching artefacts generated during reverse transcription, two different RTases, MMLV-derived RTase (Thermo Scientific) and AMV-derived RTase (New England Biolabs), are used in parallel reverse transcription experiments^24^,^29^.

cDNA from each *D. melanogaster* stage, and from the *D. plexippus* thorax and head is mixed in equal amounts, respectively, before RT–PCR experiments. The *trans*-splicing-specific oligonucleotide primers for *D. melanogaster* and *D. plexippus* are designed using Primer Premier 5 (ref. 57; Supplementary Table 3). RT–PCR reactions are performed using KOD FX (Toyobo) DNA polymerase for 50 cycles. *rp49* (a gene of ribosomal protein L32e family) in *D. melanogaster* and glyceraldehyde-3-phosphate dehydrogenase gene (DPOGS215460-TA) and actin gene (DPOGS207542-TA) in *D. plexippus* are used as controls. The amplified *trans*-spliced products are extracted from an agarose gel with a gel extraction kit (OMEGA) and are sequenced from both ends by Sanger sequencing. Only the candidates that are validated by RT–PCR using both MMLV-derived RTase and AMV-derived RTase are considered to be true positives for *trans*-splicing events.

### Pairwise comparison of *trans*-spliced transcripts

*Trans*-spliced coding transcripts are first translated into protein sequences. Pairwise comparisons are then performed between different species using BLASTP software (version 2.2.21). The donor and acceptor segments are compared separately. Events with both the donor and the acceptor reaching the threshold (*E*-value of 1E-5, coverage of 60% and identity of 30%) are considered to be conserved *trans*-splicing events. As both the donor and acceptor segments become incorporated into *trans*-spliced products, the criteria are designed to exclude cases of a partial match by either the donor or the acceptor segment alone.

### *Mod(mdg4)*-like gene identification in additional species

The PFAM profile hidden Markov models^58^ for BTB and FLYWCH domains are downloaded from the PFAM database (http://pfam.xfam.org/). Hmmsearch^59^ (HMMer package version3.1b1) is used to search for their hits in all annotated genes from *T. castaneum*, *A. mellifera*, *C. floridanus* and *D. pulex*, with the parameters ‘-E 1e-5 --incE 1e-5 --domE 1e-5 --incdomE 1e-5'. Genes that exceed these thresholds and have the correct configuration with the BTB domain upstream to the FLYWCH domain, are identified as *mod(mdg4)*-like genes.

### Phylogenetic analysis of *mod(mdg4)*-like proteins

The common N-terminus of *mod(mdg4)* proteins from the six insect species *D. melanogaster*, *B .mori*, *A. aegypti*, *D. plexippus, A. gambiae*^17^, *P. xylostella* and *D. pulex* are aligned using the PFAM profile hidden Markov model for BTB domain, and HMMalign^59^ (HMMer package version 3.1b1). Multiple alignments for the BTB domain regions are shown in Fig. 4a,b.

Hmmsearch^59^ is used to search for hits of FLYWCH domain in the variable C-terminus of *mod(mdg4)* proteins from the six species with the *E*-value 1e-5. The identified FLYWCH domain regions are isolated and aligned using the pHMM FLYWCH.hmm and HMMalign. Terminal tails of non-aligned residues are trimmed, and unambiguous sequence alignments are subsequently subjected to phylogenetic analysis. The ML analysis is performed with the programme PhyML^60^ (version 3.1) using the default parameters (amino-acid substitution model: LG; shape parameter: estimated gamma distribution). The *D. pulex* FLYWCH domain sequence is used as the outgroup. The ML analysis is initiated with a BIONJ tree, and tree topology is searched by the Nearest-Neighbour Interchanges method. The Shimodaira–Hasegawa-like approximate likelihood ratio test is used for the branch supports. The phylogenetic tree is visualized with the programme Figtree^61^.

### *Trans*-spliced products resembling canonical transcripts

*Trans*-spliced coding transcripts are first translated into protein sequences. Pairwise comparisons and alignments using the BLASTP (version 2.2.21) programme are performed between the *trans*-spliced products and the canonical gene products from each of the other 13 species, including *C. pipiens*, *A. aegypti, A. gambiae*, *D. melanogaster*, *P. xylostella*, *B. mori, H. melpomene*, *D. plexippus*, *T. castaneum*, *A. mellifera*, *A. echinatior*, *C. floridanus*, *A. pisum* and *D. pulex*. A ‘homologous' *trans*-splicing event is defined as when an alignment between a *trans*-splicing product and a canonical protein reaches the threshold (*E*-value of 1E-5, coverage of 90% and identity of 50%). The stringent criteria are used to exclude partial homologues between *trans*-spliced products and canonical gene products.

## Additional information

**How to cite this article:** Kong, Y. *et al*. The evolutionary landscape of intergenic *trans*-splicing events in insects. *Nat. Commun.* 6:8734 doi: 10.1038/ncomms9734 (2015).

## Supplementary Material

Supplementary Information Supplementary Figures 1-7, Supplementary Tables 1-6 and Supplementary References

Supplementary Data 1 Information of trans-splicing events

Supplementary Data 2 Protein products of trans-splicing events

Supplementary Data 3 List of trans-spliced coding transcripts homologous to canonical cis-splicing genes from different species

## Figures and Tables

**Table 1:** Pairwise analysis of homologue between *trans*-spliced coding transcripts and canonical coding genes using BLASTP.

Number*	**Canonical coding gene**													
	**Cpi**	**Aae**	**Aga**	**Dme**	**Pxy**	**Bmo**	**Hme**	**Dpl**	**Tca**	**Ame**	**Aec**	**Cfl**	**Api**	**Dpu**
*Trans*-spliced coding transcript														
Aae	32	−	37	24	9	8	11	11	17	17	16	17	14	12
Dme	7	11	8	−	3	6	8	4	10	7	8	15	8	7
Bmo	3	3	1	2	4	−	3	7	4	2	2	2	2	1
Dpl	2	2	0	0	7	4	8	−	0	2	0	0	1	2
Tca	0	0	2	1	1	0	0	2	−	2	1	1	1	0
Ame	2	1	3	4	0	1	0	1	1	−	7	5	1	2
Cfl	0	0	0	0	0	0	1	0	1	1	2	−	0	0
Api	7	10	12	10	5	5	3	11	17	9	14	13	−	10

Aga, *A. gambiae*; Aec, *A. echinatior;* Aae, *A. aegypti;* Ame, *A. mellifera*; Api, *A. pisum*; Bmo, *B. mori*; Cfl, *C. floridanus*; Cpi, *C. pipiens*; Dpu, *D. pulex*; Dme, *D. melanogaster*; Dpl, *D. plexippus*; Hme, *H. melpomene*; Pxy, *P. xylostella*; Tca, *T. castaneum.*

^*^Number of non-*mod trans*-spliced coding transcripts that found at least one match to canonical coding gene in other species. The threshold for a match requires 50% identity and 90% coverage between a *trans*-spliced transcript and a canonical coding gene of other species.
