# Supplementary material for: The evolutionary landscape of intergenic trans-splicing events in insects
Source: Nat Commun. 2015 Nov 2;6:8734. doi: 10.1038/ncomms9734 (PMC4667647; doi:10.1038/ncomms9734)
Supplement: Supplementary Information — Supplementary Figures 1-7, Supplementary Tables 1-6 and Supplementary References [file ncomms9734-s1.pdf]

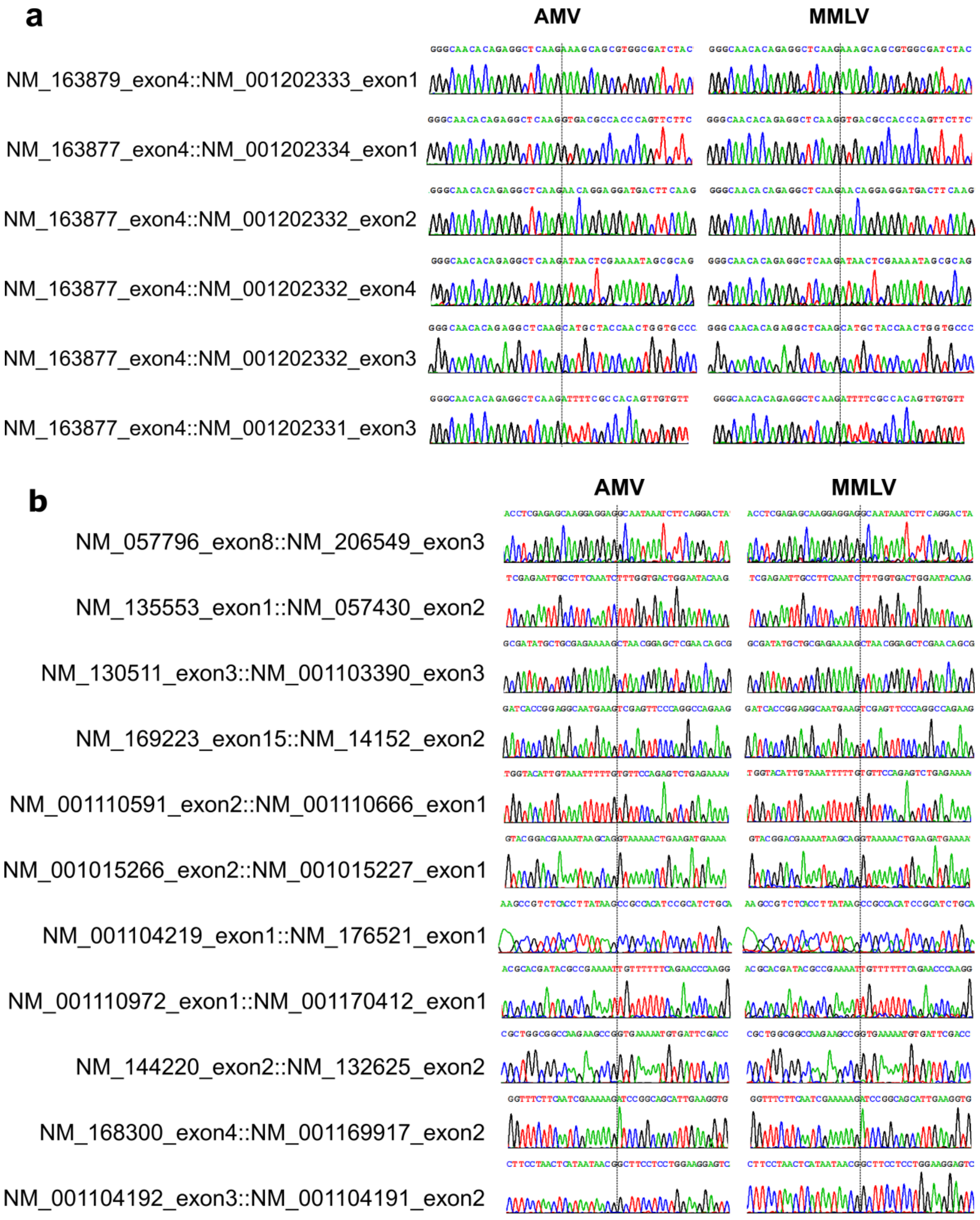
a
AMV
MMLV
NM_163879_exon4::NM_001202333_exon1
NM_163877_exon4::NM_001202334_exon1
NM_163877_exon4::NM_001202332_exon2
NM_163877_exon4::NM_001202332_exon4
NM_163877_exon4::NM_001202332_exon3
NM_163877_exon4::NM_001202331_exon3
b
AMV
MMLV
NM_057796_exon8::NM_206549_exon3
NM_135553_exon1::NM_057430_exon2
NM_130511_exon3::NM_001103390_exon3
NM_169223_exon15::NM_14152_exon2
NM_001110591_exon2::NM_001110666_exon1
NM_001015266_exon2::NM_001015227_exon1
NM_001104219_exon1::NM_176521_exon1
NM_001110972_exon1::NM_001170412_exon1
NM_144220_exon2::NM_132625_exon2
NM_168300_exon4::NM_001169917_exon2
NM_001104192_exon3::NM_001104191_exon2

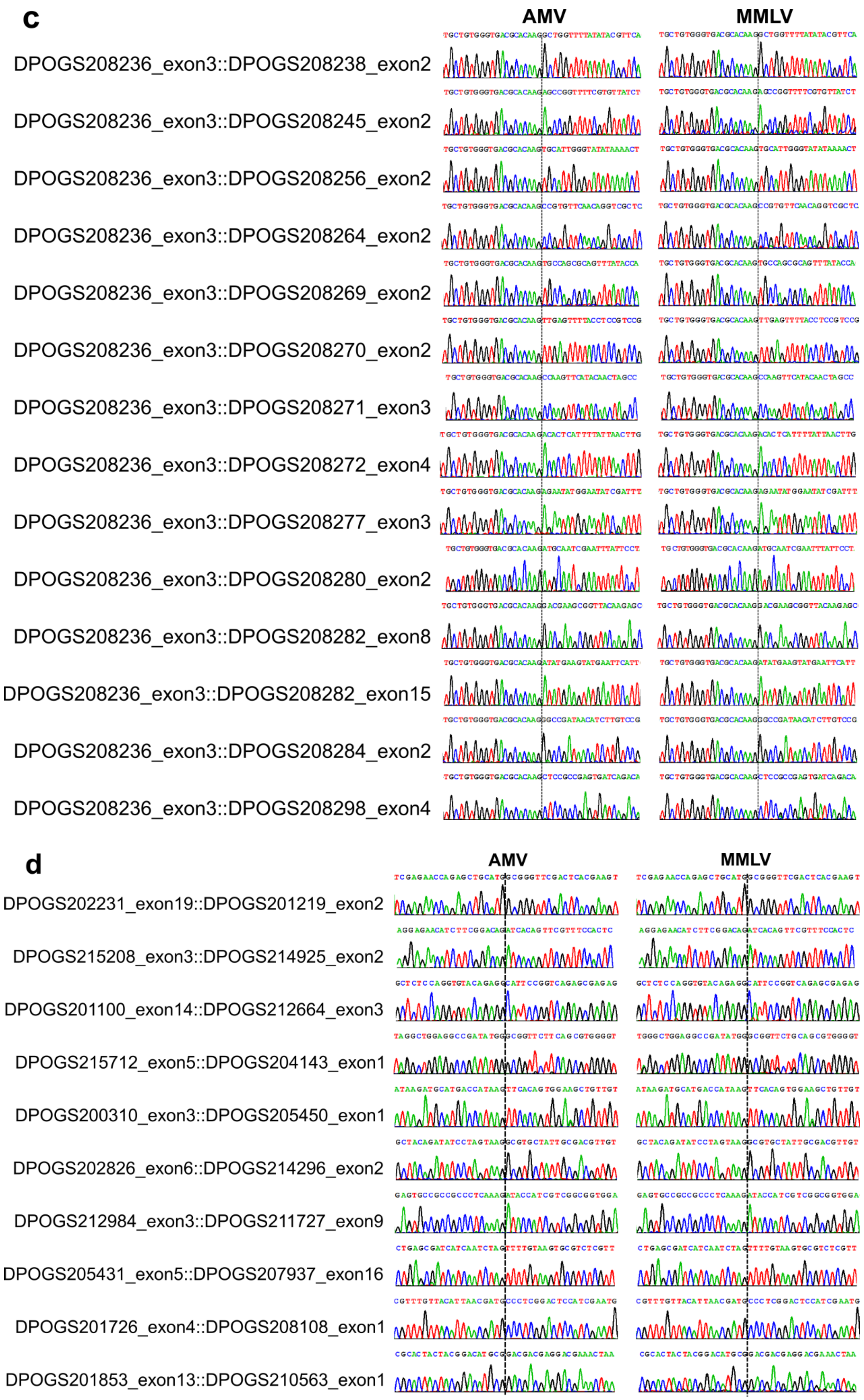
c
AMV
MMLV
DPOGS208236_exon3::DPOGS208238_exon2
DPOGS208236_exon3::DPOGS208245_exon2
DPOGS208236_exon3::DPOGS208256_exon2
DPOGS208236_exon3::DPOGS208264_exon2
DPOGS208236_exon3::DPOGS208269_exon2
DPOGS208236_exon3::DPOGS208270_exon2
DPOGS208236_exon3::DPOGS208271_exon3
DPOGS208236_exon3::DPOGS208272_exon4
DPOGS208236_exon3::DPOGS208277_exon3
DPOGS208236_exon3::DPOGS208280_exon2
DPOGS208236_exon3::DPOGS208282_exon8
DPOGS208236_exon3::DPOGS208282_exon15
DPOGS208236_exon3::DPOGS208284_exon2
DPOGS208236_exon3::DPOGS208298_exon4
d
AMV
MMLV
DPOGS202231_exon19::DPOGS201219_exon2
DPOGS215208_exon3::DPOGS214925_exon2
DPOGS201100_exon14::DPOGS212664_exon3
DPOGS215712_exon5::DPOGS204143_exon1
DPOGS200310_exon3::DPOGS205450_exon1
DPOGS202826_exon6::DPOGS214296_exon2
DPOGS212984_exon3::DPOGS211727_exon9
DPOGS205431_exon5::DPOGS207937_exon16
DPOGS201726_exon4::DPOGS208108_exon1
DPOGS201853_exon13::DPOGS210563_exon1

Supplementary Figure 1. Validation of *trans*-splicing events by RT-PCR experiments and Sanger sequencing. RT-PCR analyses were performed in parallel with two different reverse transcriptases, Avian Myeloblastosis Virus (AMV)-derived RTase and Moloney Murine Leukemia Virus (MMLV)-derived RTase. Validated t*rans*-splicing events by both reverse transcriptases are shown. a, Six *mod(mdg4*) *trans*-spliced products from *D. melanogaster.* b, Eleven non-*mod trans*-spliced products from *D. melanogaster.* c, Fourteen *mod(mdg4*) *trans*-spliced products from *D. plexippus*. d, Ten non-*mod trans*-spliced products from *D. plexippus.* Sequence primers are listed in Supplementary Table 3. Splice junctions are marked with vertical dashed lines.

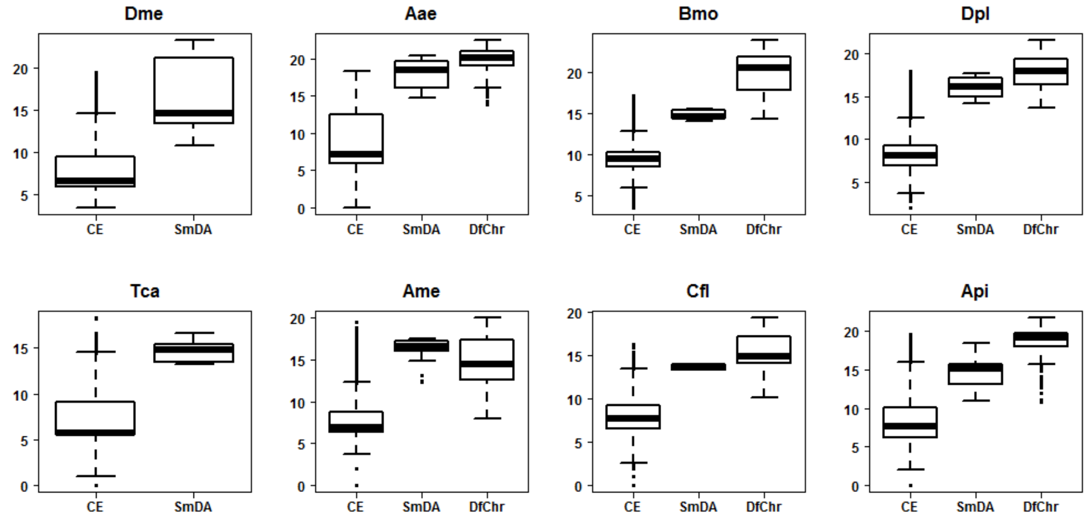

Supplementary Figure 2. Distances between exons of canonical genes, and between donor and acceptor genes of *trans*-splicing events. Box plots show the distances (log2 transformed) between canonical exons, and between donor and acceptor genes of *trans*-splicing events from the eight species. The bottom and top of the box represent the first and third quartiles, and the band inside the box the second quartile (the median). The whiskers extend 1.5 interquartile range (third quartile minus first quartile) upward from the third quartile, and downward from the first quartile. Outliers are marked with cross. CE, canonical exons. SmDA, donor and acceptor genes on the same chromosome/scaffold with donor upstream of acceptor. DfChr, donor and acceptor genes on different chromosomes/scaffolds; The distance for DfChr was computed assuming donor and acceptor scaffolds are located on the same chromosome and take a donor-upstream-acceptor configuration. Dme, *D. melanogaster*; Aae, *A. aegypti;* Bmo, *B. mori*; Dpl, *D. plexippus*; Tca, *T. castaneum*; Ame, *A. mellifera*; Cfl, *C. floridanus*; Api, *A. pisum*.

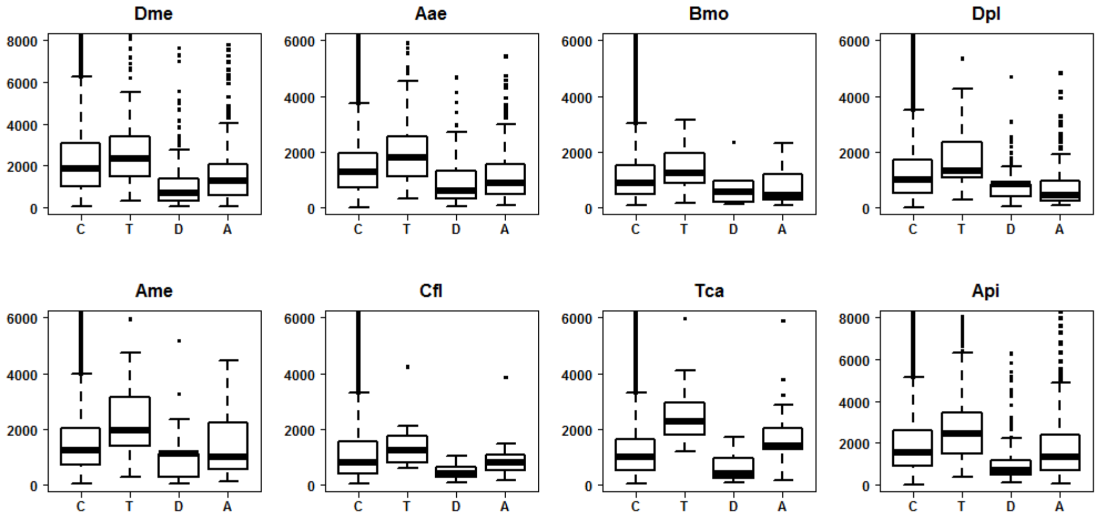

Supplementary Figure 3. Length of canonical transcripts, *trans*-spliced products, and donor and acceptor segments of *trans*-splicing events. Box plots show the length of canonical transcripts (C), *trans*-spliced products (T), and donor (D) and acceptor (A) segments of *trans*-splicing events in eight species. The parameters used in the box plots are the same as those of Supplementary Fig. 2. Dme, *D. melanogaster*; Aae, *A. aegypti;* Bmo, *B. mori*; Dpl, *D. plexippus*; Tca, *T. castaneum*; Ame, *A. mellifera*; Cfl, *C. floridanus*; Api, *A. pisum*.

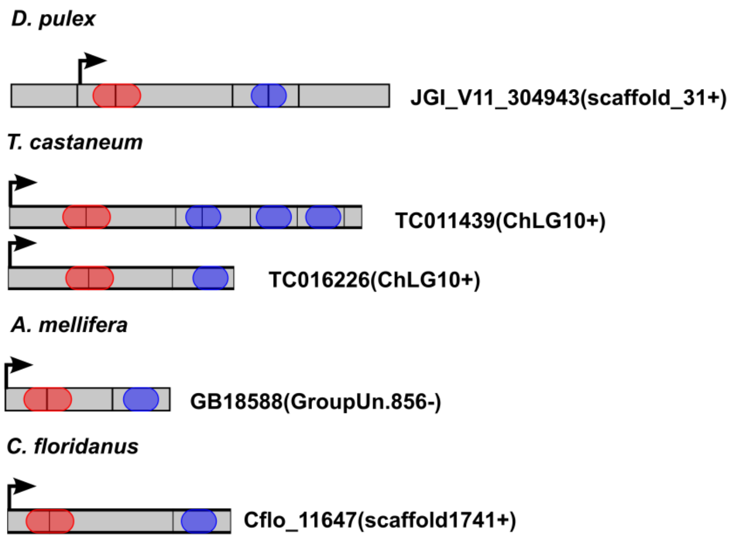

Supplementary Figure 4. Schematic diagram of the *mod(mdg4)*-like gene in *D. pulex*, *C. floridanus*, *A. mellifera* and *T. castaneum*. Gene name and location are indicated at the right of the schematic diagram. Black arrow, translation initiation site. Grey box, exon. Red oval, BTB domain. Blue oval, FLYWCH domain.

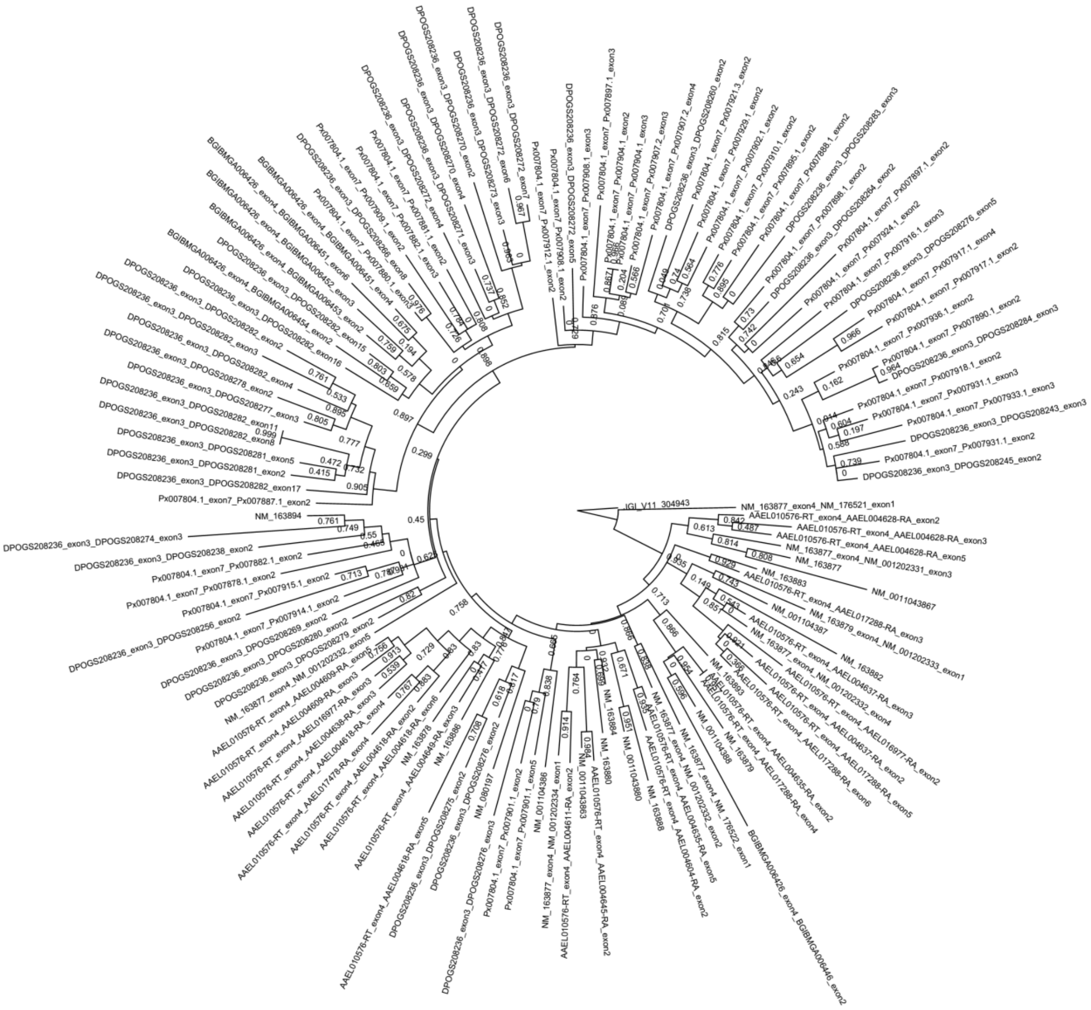

Supplementary Figure 5. Phylogenetic analysis of *mod(mdg4)* C-terminus FLYWCH domains using Maximum Likelihood. The maximum likelihood (ML) analysis was performed by the program PhyML[1] (version 3.1). The ratio of Shimodaira-Hasegawa-like approximate likelihood-ratio test (SH-aLRT) is labeled on the branch node.

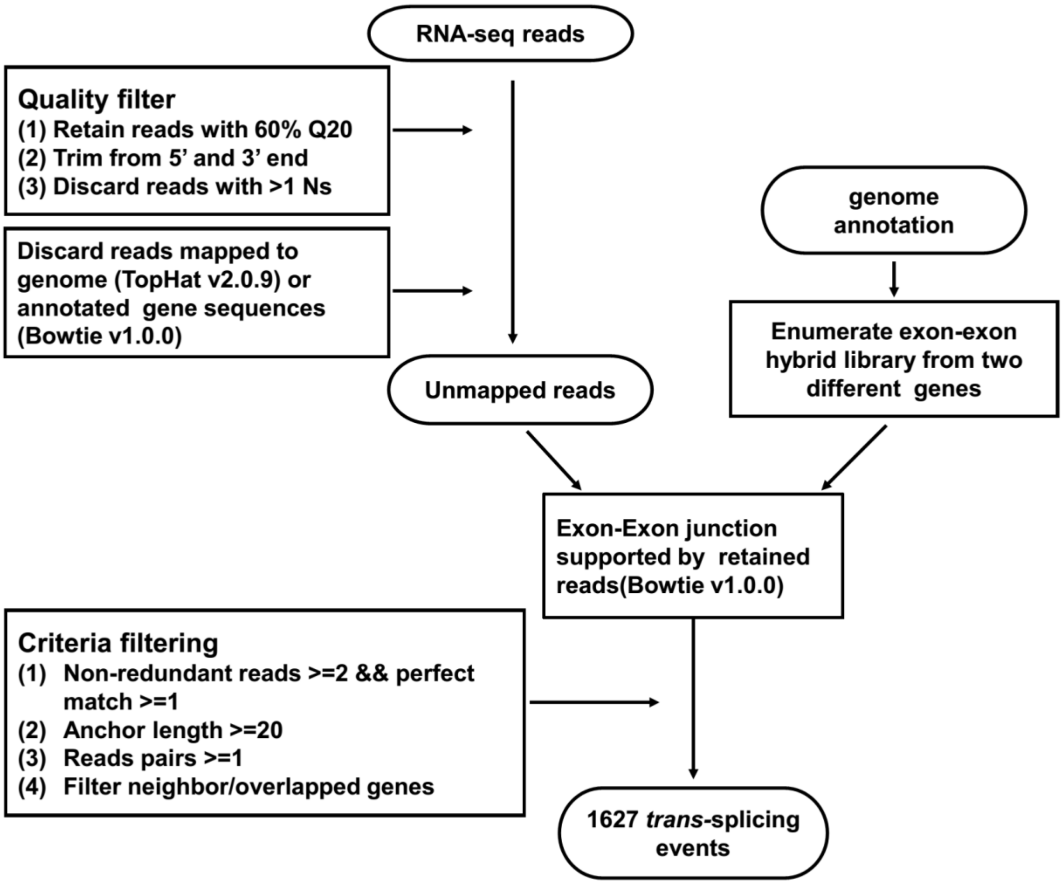

Supplementary Figure 6. Bioinformatics pipeline for *trans*-splicing identification. The ovals indicate the major income and outcome. The boxes indicate the processing methods, softwares and parameters used in the workflow.

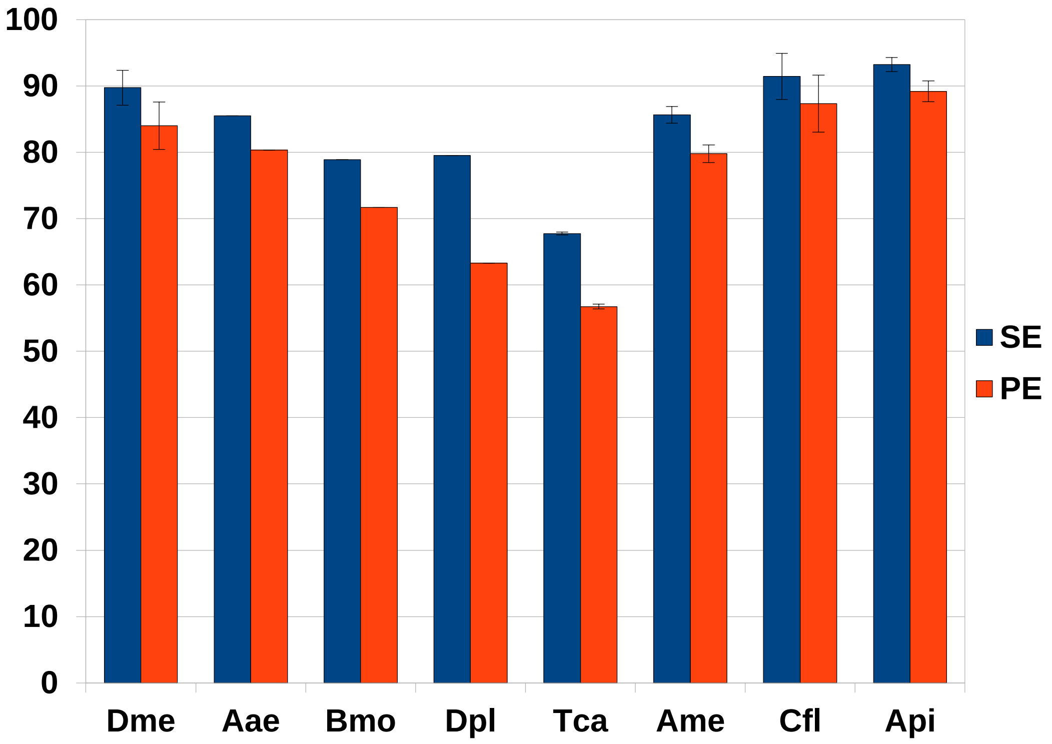

Supplementary Figure 7. Mapping rate of single-end and paired-end RNA-seq reads for the eight insect species. For each species, the RNA-seq reads were aligned to the reference genome with TopHat (v2.0.9) with the default parameters. The paired-end RNA-seq data used for Bmo were obtained from NCBI-SRA database with accession number SRR1805030. The error bars indicate the s.d. if multiple SRA runs are provided. SE: single-end reads. PE: paired-end reads. Dme, *D. melanogaster*; Aae, *A. aegypti;* Bmo, *B. mori*; Dpl, *D. plexippus*; Tca, *T. castaneum*; Ame, *A. mellifera*; Cfl, *C. floridanus*; Api, *A. pisum*.

gene	*Trans*-splicing	Junction	Donor	Acceptor	Reads	Anchor	Perfect	PE
**Dme *mod(mdg4)***	NM_163877_exon4::NM_001202332_exon5	chr3R-17200782::chr3R+17193632	mod(mdg4)|NM_163877	CG42775|NM_001202332	2268/117	70/70	116	1172
	NM_163879_exon4::NM_001202333_exon1	chr3R-17200782::chr3R+17194102	mod(mdg4)|NM_163879	CG42776|NM_001202333	1559/113	69/70	108	1202
	NM_163877_exon4::NM_176521_exon1	chr3R-17200782::chr3R+17186112	mod(mdg4)|NM_163877	mod(mdg4)|NM_176521	871/64	70/70	60	1270
	NM_163877_exon4::NM_176522_exon1	chr3R-17200782::chr3R+17187120	mod(mdg4)|NM_163877	mod(mdg4)|NM_176522	468/89	70/70	79	398
	NM_163877_exon4::NM_001202334_exon1	chr3R-17200782::chr3R+17195184	mod(mdg4)|NM_163877	CG42777|NM_001202334	435/87	69/70	80	380
	NM_163877_exon4::NM_001202332_exon4	chr3R-17200782::chr3R+17193085	mod(mdg4)|NM_163877	CG42775|NM_001202332	229/75	68/70	65	132
	NM_163877_exon4::NM_001202332_exon2	chr3R-17200782::chr3R+17191726	mod(mdg4)|NM_163877	CG42775|NM_001202332	188/65	68/70	55	139
	NM_163877_exon4::NM_001202332_exon3	chr3R-17200782::chr3R+17192172	mod(mdg4)|NM_163877	CG42775|NM_001202332	69/40	68/70	32	55
	NM_163877_exon4::NM_001202331_exon3	chr3R-17200782::chr3R+17192696	mod(mdg4)|NM_163877	CG42774|NM_001202331	38/28	69/70	20	19
**Bmo *mod(mdg4)***	BGIBMGA006426_exon4::BGIBMGA006451_exon6	nscaf2853-891737::nscaf2853+533282	BGIBMGA006426	BGIBMGA006451	44/16	67/68	11	-
	BGIBMGA006426_exon4::BGIBMGA006445_exon2	nscaf2853-891737::nscaf2853+318730	BGIBMGA006426	BGIBMGA006445	21/6	68/47	4	-
	BGIBMGA006426_exon4::BGIBMGA006453_exon2	nscaf2853-891737::nscaf2853+566158	BGIBMGA006426	BGIBMGA006453	8/2	53/57	2	-
	BGIBMGA006426_exon4::BGIBMGA006451_exon4	nscaf2853-891737::nscaf2853+529907	BGIBMGA006426	BGIBMGA006451	6/2	43/37	2	-
	BGIBMGA006426_exon4::BGIBMGA006454_exon2	nscaf2853-891737::nscaf2853+571412	BGIBMGA006426	BGIBMGA006454	5/3	69/29	1	-
	BGIBMGA006426_exon4::BGIBMGA006452_exon3	nscaf2853-891737::nscaf2853+560780	BGIBMGA006426	BGIBMGA006452	4/2	38/67	1	-
	BGIBMGA006426_exon4::BGIBMGA006446_exon2	nscaf2853-891737::nscaf2853+349483	BGIBMGA006426	BGIBMGA006446	2/2	39/28	1	-
**Bmo non-*mod***	BGIBMGA013060_exon2::BGIBMGA002217_exon2	nscaf3058+6912054::nscaf2279-11005	BGIBMGA013060	BGIBMGA002217	58/25	69/65	20	-
	BGIBMGA013935_exon1::BGIBMGA013937_exon2	nscaf3099-393215::nscaf3099-371739	BGIBMGA013935	BGIBMGA013937	4/3	34/27	2	-
	BGIBMGA007426_exon1::BGIBMGA008579_exon27	nscaf2883+1908868::nscaf2903-881406	BGIBMGA007426	BGIBMGA008579	10/6	56/50	5	-

	BGIBMGA008294_exon4::SIBSBM001135_exon2	nscaf2902-10945528::nscaf3058+3507290	BGIBMGA008294	SIBSBM001135	13/8	66/68	7	-
	BGIBMGA005174_exon4::BGIBMGA004935_exon2	nscaf2823+4295037::nscaf2822-967220	BGIBMGA005174	BGIBMGA004935	12/5	55/64	5	-
	BGIBMGA012150_exon1::BGIBMGA002352_exon2	nscaf3035-1120363::nscaf2366+1098	BGIBMGA012150	BGIBMGA002352	6/4	62/22	4	-
	SIBSBM000695_exon6::BGIBMGA004353_exon2	nscaf2789+1576593::nscaf2793+7447	SIBSBM000695	BGIBMGA004353	5/3	51/65	2	-
	BGIBMGA014566_exon8::SIBSBM000567_exon4	scaffold838-6767::nscaf2176-502953	BGIBMGA014566	SIBSBM000567	6/4	56/52	3	-
	BGIBMGA014447_exon2::BGIBMGA014500_exon2	scaffold665-19260::scaffold748+12798	BGIBMGA014447	BGIBMGA014500	4/3	23/66	2	-

Supplementary Table 1. Previously confirmed *trans*-splicing events identified in this study. In total, we identified 25 *trans*-splicing events confirmed by previous studies, including 9 *mod(mdg4)* with exons located on the opposite strands in *D. melanogaster* [2,3], 7 *mod(mdg4)* and 9 non-*mod* in *B. mori*[4]**.** Reads: all supporting reads and non-redundant supporting reads; Anchor: arm length of reads covering the junction site; PE: paired-end supporting reads; Perfect: supporting reads with no mismatch; Dme, *D. melanogaster*; Bmo, *B. mori*.

**Species**	**Donor**	**Acceptor**	**Reads**	**Anchor**	**PE**	**Gene location**
**Dme**	NM_057796_exon8	NM_206549_exon3	6/5	42/59	15	chr3R(+)/chr3R(-)
**Dme**	NM_135553_exon1	NM_057430_exon2	4/4	63/38	5	chr2L(-)/chr2L(+)
**Dme**	NM_130511_exon3	NM_001103390_exon3	12/12	64/58	265	chrX(-)/chrX(-)
**Dme**	NM_169223_exon15	NM_141542_exon2	21/17	68/68	60	chr3R(-)/chr3R(-)
**Dme**	NM_001110591_exon2	NM_001110666_exon1	32/22	69/65	45	chrU(+)/chrU(+)
**Dme**	NM_001015266_exon2	NM_001015227_exon1	25/18	65/70	39	chrU(-)/chr3LHet(-)
**Dme**	NM_001104219_exon1	NM_176521_exon1	3/2	64/70	26	chr3R(-)/chr3R(+)
**Dme**	NM_001110972_exon1	NM_001170412_exon1	17/14	69/70	21	chr3RHet(+)/chrU(+)
**Dme**	NM_144220_exon2	NM_132625_exon2	7/6	60/57	307	chrX(-)/chrX(-)
**Dme**	NM_168300_exon4	NM_001169917_exon2	9/8	59/69	303	chr3L(-)/chr3L(-)
**Dme**	NM_001104192_exon3	NM_001104191_exon2	8/7	63/52	41	chr3L(-)/chr3L(-)
**Dpl**	DPOGS202231_exon19	DPOGS201219_exon2	912/98	70/70	1591	DPSCF300149(+)/DPSCF301271(-)
**Dpl**	DPOGS215208_exon3	DPOGS214925_exon2	136/54	70/70	396	DPSCF300143(+)/DPSCF300625(+)
**Dpl**	DPOGS201100_exon14	DPOGS212664_exon3	92/26	70/49	221	DPSCF300137(-)/DPSCF300198(-)
**Dpl**	DPOGS215712_exon5	DPOGS204143_exon1	38/22	70/68	74	DPSCF300041(+)/DPSCF302911(+)
**Dpl**	DPOGS200310_exon3	DPOGS205450_exon1	124/34	68/70	304	DPSCF300026(-)/DPSCF301562(+)
**Dpl**	DPOGS202826_exon6	DPOGS214296_exon2	282/56	70/68	567	DPSCF300018(+)/DPSCF301889(-)
**Dpl**	DPOGS212984_exon3	DPOGS211727_exon9	215/66	70/70	1542	DPSCF300792(+)/DPSCF300239(+)
**Dpl**	DPOGS205431_exon5	DPOGS207937_exon16	12/8	51/62	73	DPSCF300925(+)/DPSCF300090(-)
**Dpl**	DPOGS201726_exon4	DPOGS208108_exon1	86/36	69/60	237	DPSCF300269(+)/DPSCF303241(+)
**Dpl**	DPOGS201853_exon13	DPOGS210563_exon1	230/48	69/70	421	DPSCF300191(-)/DPSCF301605(+)

Supplementary Table 2. Verification of non-*mod trans*-splicing events by RT-PCR and Sanger sequencing. Information of the 11 non-*mod trans*-splicing events from *D. melanogaster* and 10 non-*mod trans*-splicing events from *D. plexippus* were summarized. These *trans*-splicing events were RT-PCR amplified with Sanger sequencing confirmation**.** Reads: all supporting reads and non-redundant supporting reads; Anchor: arm length of reads covering the junction site; PE: paired-end supporting reads; Dme, *D. melanogaster*; Dpl, *D. plexippus*.

Species	Gene	5' primer	3' primer	Product
**Dme**	NM_163879_exon4::NM_001202333_exon1	GTCAAGCAACAGTCCCAGAAC	CCACCTTGGCAATCGTCTC	358
**Dme**	NM_163877_exon4::NM_001202334_exon1	GTCAAGCAACAGTCCCAGAAC	GGAAGTTGGCTTTAGTCACAG	378
**Dme**	NM_163877_exon4::NM_001202332_exon2	GTCAAGCAACAGTCCCAGAAC	ACGCACTCCCAGTAGCACA	281
**Dme**	NM_163877_exon4::NM_001202332_exon4	GTCAAGCAACAGTCCCAGAAC	GTCAACCACGCTAATGTCC	345
**Dme**	NM_163877_exon4::NM_001202332_exon3	GTCAAGCAACAGTCCCAGAAC	TGAAAGCCCAAAACCAAAT	484
**Dme**	NM_163877_exon4::NM_001202331_exon3	GTCAAGCAACAGTCCCAGAAC	GCCTCATCCGCACTTTGT	253
**Dme**	NM_057796_exon8::NM_206549_exon3	GTTTACGTTGCCCAAGTGTC	GGTCCTGCTCGTTGTAGTCC	204
**Dme**	NM_135553_exon1::NM_057430_exon2	CTATCGCAAGTTAGTGTCCCATT	CGTTCTCGTCAACCTTGTAGTATTT	504
**Dme**	NM_130511_exon3::NM_001103390_exon3	ATTGGCAGGAGCGGTGTC	GCCTGATGGCTGGGAGAAC	304
**Dme**	NM_169223_exon15::NM_141542_exon2	GAAATCACGCTCGGATAGCA	GGAATCGGAGGGATAGTGC	301
**Dme**	NM_001110591_exon2::NM_001110666_exon1	CACGGCAGTGTATTTATTTCC	GGCGTAAGCCTTGACCAT	497
**Dme**	NM_001015266_exon2::NM_001015227_exon1	CCCTTTGGGTACTAGCAAACACG	GAGTGGCCTTGGGATCTCCTTTA	432
**Dme**	NM_001104219_exon1::NM_176521_exon1	AAATCGACGGAGCAACAA	CGGTAGCAGCACTGAAACA	318
**Dme**	NM_001110972_exon1::NM_001170412_exon1	CCGTCAATGAAGGCTGTT	CTCTAATACGTCGTTTGCTGT	270
**Dme**	NM_144220_exon2::NM_132625_exon2	CGCCGAGAATCTGCTAATC	GTGTCCGTAAATCCCACGA	306
**Dme**	NM_168300_exon4::NM_001169917_exon2	AACGGGCTGGGATTGGTC	GTTGATGGTGGATCGGCTA	407
**Dme**	NM_001104192_exon3::NM_001104191_exon2	ACAGCCTGACCCATCCCTA	GCGTTCGCACCAAACAAG	261
**Dme**	*rp49*	GCACTTCATCCGCCACC	TCTCGCCGCAGTAAACG	242
**Dpl**	DPOGS208236_exon3::DPOGS208238_exon2	GGCACGAATAATGATGAAAC	CAACGCTACTTGGCACCT	282
**Dpl**	DPOGS208236_exon3::DPOGS208245_exon2	TAAGCCCTCCTCAACTCCAG	ACGACTGCCACTCCAACG	460
**Dpl**	DPOGS208236_exon3::DPOGS208256_exon2	TAAGCCCTCCTCAACTCC	CGCAATAGAATGTAAACCCT	443
**Dpl**	DPOGS208236_exon3::DPOGS208264_exon2	GCACGAATAATGATGAAACG	GTGTAGACGTGGGCCTTG	274
**Dpl**	DPOGS208236_exon3::DPOGS208269_exon2	CGACGACGGCACGAATAA	GATAACATCAGCACGACAACCA	289
**Dpl**	DPOGS208236_exon3::DPOGS208270_exon2	ACGGCACGAATAATGATGAAAC	TTTGTCCGTGATAATGAGATGG	293
**Dpl**	DPOGS208236_exon3::DPOGS208271_exon3	AACTTTGGCGAAGATGACTC	TGGTATGGGTACTTGGGATT	368
**Dpl**	DPOGS208236_exon3::DPOGS208272_exon4	CGAACTTTGGCGAAGATGACT	ACCGTTGTGGTTGAGGCTGT	314
**Dpl**	DPOGS208236_exon3::DPOGS208277_exon3	CTTTGGCGAAGATGACTC	AACATAGTGCGGTTTAGTGT	319
**Dpl**	DPOGS208236_exon3::DPOGS208280_exon2	GAGGGTGGTGCTGTGGGTGA	TTTGTCCGTCCTGGGTTCGA	250
**Dpl**	DPOGS208236_exon3::DPOGS208282_exon8	TCACGATACCAGACGAGGAT	TTACTTGGCTCCGAATCACT	313
**Dpl**	DPOGS208236_exon3::DPOGS208282_exon15	ATTTGTCACGATACCAGACGAGGAT	CGGGCTCTACACTGACTGGATTT	363
**Dpl**	DPOGS208236_exon3::DPOGS208284_exon2	ACGATACCAGACGAGGATG	GTACGGTCATATCACAAGGTC	394
**Dpl**	DPOGS208236_exon3::DPOGS208298_exon4	TGTCACGATACCAGACGAGGAT	CCTGTTCACCATTGCCAGAT	506
**Dpl**	DPOGS202231_exon19::DPOGS201219_exon2	TCACGAGGACGATGAAAGC	ATCCGCTCCGAGGACTTG	273
**Dpl**	DPOGS215208_exon3::DPOGS214925_exon2	CTACCCGGAGTTCAGACATAA	TCCTTCACATCTTTCGCTTC	405
**Dpl**	DPOGS201100_exon14::DPOGS212664_exon3	AGACAGCTTGGCAGACATCTC	TCTCAGTAGACTGGTGAATGGC	223
**Dpl**	DPOGS215712_exon5::DPOGS204143_exon1	GTGCGACCCGTCCAAAGACT	CAAGCGGTCACGTTATCATTCT	302
**Dpl**	DPOGS200310_exon3::DPOGS205450_exon1	GGATGGGTCTCGGTTGGAAT	TGAGGAGTTGCTGCGTCAGG	300
**Dpl**	DPOGS202826_exon6::DPOGS214296_exon2	CGATAGATTCGGCTGGTTT	TGATAGAAGTGCGGTTGTGATA	459
**Dpl**	DPOGS212984_exon3::DPOGS211727_exon9	AAGACGGACGAGGGATACGG	GGGTTCCAGGGCTCCAAAC	276
**Dpl**	DPOGS205431_exon5::DPOGS207937_exon16	ATGCCGCCTTCAAATCAG	TGCTGTACGATGCCATAACG	482

**Dpl**	DPOGS201726_exon4::DPOGS208108_exon1	CCACGAGAAGGTGCTGTTC	CTGTCGGTTTGCCTGTCC	336
**Dpl**	DPOGS201853_exon13::DPOGS210563_exon1	ACCGGAGATCACCAACATAGCG	GCGTCACGAGCGAGTTCAGC	386
**Dpl**	*Actin*(DPOGS207542-TA)	ACACCGTGCCCATCTATGAA	AGAAGGAAGGCTGGAACAGG	318
**Dpl**	*GAPDH*(DPOGS215460-TA)	TGGAAGGTGGAGCCAAGAA	GCGAGGCGGACTGTCAAAT	415

Supplementary Table 3. Primers for RT-PCR and Sanger sequencing. The 5' primers of the donor genes, 3' primers of the acceptor genes and the predicted product length are listed. The primers were designed with Primer Premier 5[5] program and synthesized in Life Technologies Company. Dme, *D. melanogaster*; Dpl, *D. plexippus*.

Species	*Trans*-splicing events	Species	*Trans*-splicing events
Dpl	DPOGS208236_exon3::DPOGS208269_exon2	Bmo	BGIBMGA006426_exon4::BGIBMGA006452_exon3
Dpl	DPOGS208236_exon3::DPOGS208277_exon3	Bmo	BGIBMGA006426_exon4::BGIBMGA006452_exon3
Dpl	DPOGS208236_exon3::DPOGS208273_exon3	Bmo	BGIBMGA006426_exon4::BGIBMGA006453_exon2
Dpl	DPOGS208236_exon3::DPOGS208282_exon17	Bmo	BGIBMGA006426_exon4::BGIBMGA006452_exon3
Dpl	DPOGS208236_exon3::DPOGS208282_exon17	Bmo	BGIBMGA006426_exon4::BGIBMGA006453_exon2
Dpl	DPOGS208236_exon3::DPOGS208282_exon17	Bmo	BGIBMGA006426_exon4::BGIBMGA006454_exon2
Dpl	DPOGS208236_exon3::DPOGS208271_exon3	Bmo	BGIBMGA006426_exon4::BGIBMGA006454_exon2
Dpl	DPOGS208236_exon3::DPOGS208280_exon2	Bmo	BGIBMGA006426_exon4::BGIBMGA006453_exon2
Dpl	DPOGS208236_exon3::DPOGS208281_exon5	Bmo	BGIBMGA006426_exon4::BGIBMGA006451_exon6
Dpl	DPOGS208236_exon3::DPOGS208281_exon5	Bmo	BGIBMGA006426_exon4::BGIBMGA006452_exon3
Dpl	DPOGS208236_exon3::DPOGS208281_exon5	Bmo	BGIBMGA006426_exon4::BGIBMGA006453_exon2
Dpl	DPOGS208236_exon3::DPOGS208281_exon5	Bmo	BGIBMGA006426_exon4::BGIBMGA006454_exon2
Dpl	DPOGS208236_exon3::DPOGS208279_exon2	Bmo	BGIBMGA006426_exon4::BGIBMGA006453_exon2
Dpl	DPOGS208236_exon3::DPOGS208264_exon2	Bmo	BGIBMGA006426_exon4::BGIBMGA006452_exon3
Dpl	DPOGS208236_exon3::DPOGS208264_exon2	Bmo	BGIBMGA006426_exon4::BGIBMGA006453_exon2
Aae	AAEL010576-RT_exon4::AAEL017288-RA_exon6	Dme	NM_163877_exon4::NM_001202332_exon3
Aae	AAEL010576-RT_exon4::AAEL017288-RA_exon6	Dme	NM_163877_exon4::NM_001202332_exon4
Aae	AAEL010576-RT_exon4::AAEL004604-RA_exon2	Dme	NM_163877_exon4::NM_001202332_exon2
Aae	AAEL010576-RT_exon4::AAEL004604-RA_exon2	Dme	NM_163877_exon4::NM_001202332_exon3
Aae	AAEL010576-RT_exon4::AAEL004604-RA_exon2	Dme	NM_163877_exon4::NM_001202332_exon5
Aae	AAEL010576-RT_exon4::AAEL004628-RA_exon5	Dme	NM_163877_exon4::NM_001202332_exon2
Aae	AAEL010576-RT_exon4::AAEL004628-RA_exon5	Dme	NM_163877_exon4::NM_001202334_exon1
Aae	AAEL010576-RT_exon4::AAEL004638-RA_exon3	Dme	NM_163877_exon4::NM_001202332_exon2
Aae	AAEL010576-RT_exon4::AAEL004645-RA_exon2	Dme	NM_163877_exon4::NM_001202332_exon5

Supplementary Table 4. Conserved *trans*-splicing event pairs from different species. Donor and acceptor segments of *trans*-splicing proteins in different species are compared separately using BLASTP software (version 2.2.21). Conserved *trans*-splicing events are defined for the protein pairs with both the donor and the acceptor reaching the threshold (E-value of 1E-5, coverage of 60%, and identity of 30%). Dme, *D. melanogaster*; Aae, *A. aegypti;* Bmo, *B. mori*; Dpl, *D. plexippus*.

Species	Donor	Acceptor	Reads	Anchor	PE	Genes Location
**Aae**	AAEL010576-RT_exon4	AAEL004601-RA_exon3	29/21	68/69	51	supercont1.488(-)/supercont1.125(+)
**Aae**	AAEL010576-RT_exon4	AAEL004604-RA_exon2	34/19	69/68	27	supercont1.488(-)/supercont1.125(+)
**Aae**	AAEL010576-RT_exon4	AAEL004609-RA_exon3	5/3	70/68	13	supercont1.488(-)/supercont1.125(+)
**Aae**	AAEL010576-RT_exon4	AAEL004609-RA_exon5	92/44	68/69	157	supercont1.488(-)/supercont1.125(+)
**Aae**	AAEL010576-RT_exon4	AAEL004610-RA_exon2	26/20	68/70	51	supercont1.488(-)/supercont1.125(+)
**Aae**	AAEL010576-RT_exon4	AAEL004611-RA_exon2	62/30	69/70	85	supercont1.488(-)/supercont1.125(+)
**Aae**	AAEL010576-RT_exon4	AAEL004611-RA_exon3	125/41	70/70	244	supercont1.488(-)/supercont1.125(+)
**Aae**	AAEL010576-RT_exon4	AAEL004614-RA_exon4	117/49	68/70	165	supercont1.488(-)/supercont1.125(+)
**Aae**	AAEL010576-RT_exon4	AAEL004614-RA_exon6	71/31	68/68	121	supercont1.488(-)/supercont1.125(+)
**Aae**	AAEL010576-RT_exon4	AAEL004618-RA_exon2	98/37	70/70	133	supercont1.488(-)/supercont1.125(+)
**Aae**	AAEL010576-RT_exon4	AAEL004618-RA_exon4	52/31	69/70	84	supercont1.488(-)/supercont1.125(+)
**Aae**	AAEL010576-RT_exon4	AAEL004618-RA_exon5	66/32	70/68	116	supercont1.488(-)/supercont1.125(+)
**Aae**	AAEL010576-RT_exon4	AAEL004618-RA_exon6	26/19	69/68	52	supercont1.488(-)/supercont1.125(+)
**Aae**	AAEL010576-RT_exon4	AAEL004628-RA_exon2	11/9	70/64	19	supercont1.488(-)/supercont1.125(+)
**Aae**	AAEL010576-RT_exon4	AAEL004628-RA_exon3	25/18	66/67	36	supercont1.488(-)/supercont1.125(+)
**Aae**	AAEL010576-RT_exon4	AAEL004628-RA_exon5	24/16	69/70	51	supercont1.488(-)/supercont1.125(+)
**Aae**	AAEL010576-RT_exon4	AAEL004635-RA_exon2	48/26	66/63	76	supercont1.488(-)/supercont1.125(+)
**Aae**	AAEL010576-RT_exon4	AAEL004635-RA_exon5	44/26	68/70	60	supercont1.488(-)/supercont1.125(+)
**Aae**	AAEL010576-RT_exon4	AAEL004635-RA_exon6	63/30	66/68	131	supercont1.488(-)/supercont1.125(+)
**Aae**	AAEL010576-RT_exon4	AAEL004637-RA_exon2	71/39	70/70	120	supercont1.488(-)/supercont1.125(+)
**Aae**	AAEL010576-RT_exon4	AAEL004637-RA_exon3	44/26	69/65	51	supercont1.488(-)/supercont1.125(+)
**Aae**	AAEL010576-RT_exon4	AAEL004638-RA_exon3	68/32	70/69	55	supercont1.488(-)/supercont1.125(+)
**Aae**	AAEL010576-RT_exon4	AAEL004644-RA_exon2	23/18	69/70	44	supercont1.488(-)/supercont1.125(+)
**Aae**	AAEL010576-RT_exon4	AAEL004644-RA_exon4	10/5	70/68	13	supercont1.488(-)/supercont1.125(+)
**Aae**	AAEL010576-RT_exon4	AAEL004645-RA_exon2	47/27	69/67	46	supercont1.488(-)/supercont1.125(+)
**Aae**	AAEL010576-RT_exon4	AAEL004649-RA_exon3	26/17	70/65	49	supercont1.488(-)/supercont1.125(+)
**Aae**	AAEL010576-RT_exon4	AAEL016977-RA_exon2	10/9	65/70	22	supercont1.488(-)/supercont1.125(+)
**Aae**	AAEL010576-RT_exon4	AAEL016977-RA_exon3	52/28	67/70	71	supercont1.488(-)/supercont1.125(+)
**Aae**	AAEL010576-RT_exon4	AAEL017026-RA_exon3	64/28	69/70	106	supercont1.488(-)/supercont1.125(+)
**Aae**	AAEL010576-RT_exon4	AAEL017288-RA_exon3	38/21	65/70	142	supercont1.488(-)/supercont1.125(+)
**Aae**	AAEL010576-RT_exon4	AAEL017288-RA_exon4	22/15	62/67	31	supercont1.488(-)/supercont1.125(+)
**Aae**	AAEL010576-RT_exon4	AAEL017288-RA_exon5	72/31	70/67	103	supercont1.488(-)/supercont1.125(+)
**Aae**	AAEL010576-RT_exon4	AAEL017288-RA_exon6	70/37	70/70	125	supercont1.488(-)/supercont1.125(+)
**Aae**	AAEL010576-RT_exon4	AAEL017478-RA_exon4	38/22	69/61	38	supercont1.488(-)/supercont1.125(+)
**Dpl**	DPOGS208236_exon3	DPOGS208238_exon2	3/2	57/46	66	DPSCF300079(-)/DPSCF300079(-)
**Dpl**	DPOGS208236_exon3	DPOGS208243_exon3	3/3	61/28	37	DPSCF300079(-)/DPSCF300079(-)
**Dpl**	DPOGS208236_exon3	DPOGS208245_exon2	4/2	24/70	7	DPSCF300079(-)/DPSCF300079(-)
**Dpl**	DPOGS208236_exon3	DPOGS208256_exon2	4/2	37/53	83	DPSCF300079(-)/DPSCF300079(-)
**Dpl**	DPOGS208236_exon3	DPOGS208260_exon2	5/4	60/69	21	DPSCF300079(-)/DPSCF300079(-)
**Dpl**	DPOGS208236_exon3	DPOGS208264_exon2	5/2	62/68	18	DPSCF300079(-)/DPSCF300079(-)

**Dpl**	DPOGS208236_exon3	DPOGS208266_exon8	6/2	45/69	271	DPSCF300079(-)/DPSCF300079(-)
**Dpl**	DPOGS208236_exon3	DPOGS208269_exon2	32/13	69/70	189	DPSCF300079(-)/DPSCF300079(+)
**Dpl**	DPOGS208236_exon3	DPOGS208270_exon2	21/12	63/69	153	DPSCF300079(-)/DPSCF300079(+)
**Dpl**	DPOGS208236_exon3	DPOGS208270_exon4	8/6	63/69	31	DPSCF300079(-)/DPSCF300079(+)
**Dpl**	DPOGS208236_exon3	DPOGS208271_exon3	26/13	65/66	91	DPSCF300079(-)/DPSCF300079(+)
**Dpl**	DPOGS208236_exon3	DPOGS208272_exon4	16/11	63/66	19	DPSCF300079(-)/DPSCF300079(+)
**Dpl**	DPOGS208236_exon3	DPOGS208272_exon5	8/6	58/64	50	DPSCF300079(-)/DPSCF300079(+)
**Dpl**	DPOGS208236_exon3	DPOGS208272_exon6	45/26	70/69	66	DPSCF300079(-)/DPSCF300079(+)
**Dpl**	DPOGS208236_exon3	DPOGS208272_exon7	10/10	70/70	21	DPSCF300079(-)/DPSCF300079(+)
**Dpl**	DPOGS208236_exon3	DPOGS208273_exon3	5/4	57/66	28	DPSCF300079(-)/DPSCF300079(+)
**Dpl**	DPOGS208236_exon3	DPOGS208274_exon3	5/4	58/69	22	DPSCF300079(-)/DPSCF300079(+)
**Dpl**	DPOGS208236_exon3	DPOGS208275_exon2	19/14	70/68	45	DPSCF300079(-)/DPSCF300079(+)
**Dpl**	DPOGS208236_exon3	DPOGS208276_exon2	15/12	60/69	74	DPSCF300079(-)/DPSCF300079(+)
**Dpl**	DPOGS208236_exon3	DPOGS208276_exon3	28/17	67/69	90	DPSCF300079(-)/DPSCF300079(+)
**Dpl**	DPOGS208236_exon3	DPOGS208276_exon5	26/14	69/70	40	DPSCF300079(-)/DPSCF300079(+)
**Dpl**	DPOGS208236_exon3	DPOGS208277_exon3	18/11	63/66	21	DPSCF300079(-)/DPSCF300079(+)
**Dpl**	DPOGS208236_exon3	DPOGS208278_exon2	6/6	69/54	17	DPSCF300079(-)/DPSCF300079(+)
**Dpl**	DPOGS208236_exon3	DPOGS208279_exon2	9/9	69/68	30	DPSCF300079(-)/DPSCF300079(+)
**Dpl**	DPOGS208236_exon3	DPOGS208280_exon2	12/10	67/66	51	DPSCF300079(-)/DPSCF300079(+)
**Dpl**	DPOGS208236_exon3	DPOGS208281_exon2	24/15	63/69	43	DPSCF300079(-)/DPSCF300079(+)
**Dpl**	DPOGS208236_exon3	DPOGS208281_exon5	6/4	36/69	12	DPSCF300079(-)/DPSCF300079(+)
**Dpl**	DPOGS208236_exon3	DPOGS208282_exon11	10/6	65/37	78	DPSCF300079(-)/DPSCF300079(+)
**Dpl**	DPOGS208236_exon3	DPOGS208282_exon15	17/9	45/69	29	DPSCF300079(-)/DPSCF300079(+)
**Dpl**	DPOGS208236_exon3	DPOGS208282_exon16	12/9	57/66	27	DPSCF300079(-)/DPSCF300079(+)
**Dpl**	DPOGS208236_exon3	DPOGS208282_exon17	9/7	68/69	44	DPSCF300079(-)/DPSCF300079(+)
**Dpl**	DPOGS208236_exon3	DPOGS208282_exon2	12/9	67/69	27	DPSCF300079(-)/DPSCF300079(+)
**Dpl**	DPOGS208236_exon3	DPOGS208282_exon3	4/3	57/67	5	DPSCF300079(-)/DPSCF300079(+)
**Dpl**	DPOGS208236_exon3	DPOGS208282_exon4	11/9	44/69	19	DPSCF300079(-)/DPSCF300079(+)
**Dpl**	DPOGS208236_exon3	DPOGS208282_exon6	4/3	63/38	2	DPSCF300079(-)/DPSCF300079(+)
**Dpl**	DPOGS208236_exon3	DPOGS208282_exon8	28/13	65/68	84	DPSCF300079(-)/DPSCF300079(+)
**Dpl**	DPOGS208236_exon3	DPOGS208283_exon3	12/8	62/69	67	DPSCF300079(-)/DPSCF300079(+)
**Dpl**	DPOGS208236_exon3	DPOGS208284_exon2	8/6	63/64	48	DPSCF300079(-)/DPSCF300079(+)
**Dpl**	DPOGS208236_exon3	DPOGS208284_exon3	5/4	67/68	33	DPSCF300079(-)/DPSCF300079(+)
**Dpl**	DPOGS208236_exon3	DPOGS208298_exon4	8/6	56/69	12	DPSCF300079(-)/DPSCF300079(+)
**Pxy**	Px007804.1_exon7	Px003882.1_exon2	342/61	68/69	426	scaffold_278(-)/scaffold_162(-)
**Pxy**	Px007804.1_exon7	Px003880.1_exon4	255/65	69/69	302	scaffold_278(-)/scaffold_162(-)
**Pxy**	Px007804.1_exon7	Px003880.1_exon3	231/64	69/69	233	scaffold_278(-)/scaffold_162(-)
**Pxy**	Px007804.1_exon7	Px003910.1_exon2	223/46	68/69	128	scaffold_278(-)/scaffold_162(-)
**Pxy**	Px007804.1_exon7	Px003882.1_exon3	195/53	69/65	201	scaffold_278(-)/scaffold_162(-)
**Pxy**	Px007804.1_exon7	Px003907.2_exon3	135/67	68/69	84	scaffold_278(-)/scaffold_162(-)
**Pxy**	Px007804.1_exon7	Px003907.2_exon4	103/40	69/67	97	scaffold_278(-)/scaffold_162(-)

**Pxy**	Px007804.1_exon7	Px003897.1_exon2	96/33	68/69	62	scaffold_278(-)/scaffold_162(-)
**Pxy**	Px007804.1_exon7	Px003912.1_exon2	67/36	69/66	71	scaffold_278(-)/scaffold_162(-)
**Pxy**	Px007804.1_exon7	Px003898.1_exon2	63/35	69/68	88	scaffold_278(-)/scaffold_162(-)
**Pxy**	Px007804.1_exon7	Px003921.3_exon2	63/33	68/68	85	scaffold_278(-)/scaffold_162(-)
**Pxy**	Px007804.1_exon7	Px003936.1_exon3	64/32	69/69	44	scaffold_278(-)/scaffold_162(-)
**Pxy**	Px007804.1_exon7	Px003924.1_exon2	47/29	69/66	73	scaffold_278(-)/scaffold_162(-)
**Pxy**	Px007804.1_exon7	Px003901.1_exon2	52/28	62/68	63	scaffold_278(-)/scaffold_162(-)
**Pxy**	Px007804.1_exon7	Px003881.1_exon2	49/26	68/69	70	scaffold_278(-)/scaffold_162(-)
**Pxy**	Px007804.1_exon7	Px003916.1_exon3	41/26	69/69	47	scaffold_278(-)/scaffold_162(-)
**Pxy**	Px007804.1_exon7	Px003908.1_exon2	44/25	68/61	73	scaffold_278(-)/scaffold_162(-)
**Pxy**	Px007804.1_exon7	Px003901.1_exon5	35/20	67/69	75	scaffold_278(-)/scaffold_162(-)
**Pxy**	Px007804.1_exon7	Px003897.1_exon3	42/25	56/69	79	scaffold_278(-)/scaffold_162(-)
**Pxy**	Px007804.1_exon7	Px003917.1_exon4	39/27	69/54	96	scaffold_278(-)/scaffold_162(-)
**Pxy**	Px007804.1_exon7	Px003917.1_exon2	34/25	69/69	40	scaffold_278(-)/scaffold_162(-)
**Pxy**	Px007804.1_exon7	Px003904.1_exon2	34/15	69/36	59	scaffold_278(-)/scaffold_162(-)
**Pxy**	Px007804.1_exon7	Px003902.1_exon2	28/20	69/65	94	scaffold_278(-)/scaffold_162(-)
**Pxy**	Px007804.1_exon7	Px003914.1_exon2	33/26	68/69	52	scaffold_278(-)/scaffold_162(-)
**Pxy**	Px007804.1_exon7	Px003890.1_exon2	34/26	69/69	93	scaffold_278(-)/scaffold_162(+)
**Pxy**	Px007804.1_exon7	Px003909.1_exon2	32/18	62/69	72	scaffold_278(-)/scaffold_162(-)
**Pxy**	Px007804.1_exon7	Px003895.1_exon2	30/20	69/54	51	scaffold_278(-)/scaffold_162(-)
**Pxy**	Px007804.1_exon7	Px003936.1_exon4	24/16	69/60	57	scaffold_278(-)/scaffold_162(-)
**Pxy**	Px007804.1_exon7	Px003929.1_exon2	26/17	62/67	57	scaffold_278(-)/scaffold_162(-)
**Pxy**	Px007804.1_exon7	Px003931.1_exon2	27/16	69/59	35	scaffold_278(-)/scaffold_162(-)
**Pxy**	Px007804.1_exon7	Px003904.1_exon3	17/13	69/65	41	scaffold_278(-)/scaffold_162(-)
**Pxy**	Px007804.1_exon7	Px003887.1_exon2	23/14	68/69	21	scaffold_278(-)/scaffold_162(+)
**Pxy**	Px007804.1_exon7	Px003888.1_exon3	22/20	69/60	91	scaffold_278(-)/scaffold_162(+)
**Pxy**	Px007804.1_exon7	Px003918.1_exon2	18/13	69/57	65	scaffold_278(-)/scaffold_162(-)
**Pxy**	Px007804.1_exon7	Px003878.1_exon2	15/9	69/50	34	scaffold_278(-)/scaffold_162(+)
**Pxy**	Px007804.1_exon7	Px003936.1_exon2	17/11	69/62	48	scaffold_278(-)/scaffold_162(-)
**Pxy**	Px007804.1_exon7	Px003908.1_exon3	15/12	68/59	67	scaffold_278(-)/scaffold_162(-)
**Pxy**	Px007804.1_exon7	Px003915.1_exon2	15/13	68/66	29	scaffold_278(-)/scaffold_162(-)
**Pxy**	Px007804.1_exon7	Px003888.1_exon2	12/10	68/59	78	scaffold_278(-)/scaffold_162(+)
**Pxy**	Px007804.1_exon7	Px003931.1_exon3	11/8	69/67	72	scaffold_278(-)/scaffold_162(-)
**Pxy**	Px007804.1_exon7	Px003933.1_exon3	7/7	40/63	13	scaffold_278(-)/scaffold_162(-)

Supplementary Table 5. New *mod(mdg4)* cases discovered in this study. Information of *mod(mdg4)* events identified in this study were shown, including 34 events in *A. aegypti*, 40 events in *D. plexippus* and 41 events in *P. xylostella*. Reads: all supporting reads and non-redundant supporting reads; Anchor: arm length of reads covering the junction site; PE: paired-end supporting reads; Aae, *A. aegypti*; Dpl, *D.*

plexippus; Pxy, *P. xylostella*.

species	*Trans*-splicing	Junction	Donor	Acceptor	Reads	Anchor	Perfect	PE
**Dme**	NM_079643_exon2::NM_079486_exon3	chr3R+11267262::chr3L+21979738	Act88F|NM_079643	Act79B|NM_079486	8/8	61/69	1	62
**Dme**	NM_079486_exon2::NM_079643_exon3	chr3L+21979375::chr3R+11267324	Act79B|NM_079486	Act88F|NM_079643	8/6	50/68	1	108
**Dme**	NM_167693_exon2::NM_057730_exon7	chrX-20087866::chrX-20101667	Sdic3|NM_167693	sw|NM_057730	8/7	67/68	7	11
**Dme**	NM_167693_exon2::NM_206798_exon8	chrX-20087866::chrX-20101670	Sdic3|NM_167693	sw|NM_206798	8/7	70/67	4	37
**Dme**	NM_079931_exon2::NM_057730_exon7	chrX-20072303::chrX-20101667	Sdic1|NM_079931	sw|NM_057730	8/7	67/68	7	12
**Dme**	NM_079931_exon2::NM_206798_exon8	chrX-20072303::chrX-20101670	Sdic1|NM_079931	sw|NM_206798	8/7	70/67	4	38
**Dme**	NM_206793_exon2::NM_057730_exon7	chrX-20095726::chrX-20101667	Sdic2|NM_206793	sw|NM_057730	8/7	67/68	7	12
**Dme**	NM_206793_exon2::NM_206798_exon8	chrX-20095726::chrX-20101670	Sdic2|NM_206793	sw|NM_206798	8/7	70/67	4	41
**Dme**	NM_206793_exon2::NM_057730_exon7	chrX-20080167::chrX-20101667	Sdic2|NM_206793	sw|NM_057730	8/7	67/68	7	12
**Dme**	NM_206793_exon2::NM_206798_exon8	chrX-20080167::chrX-20101670	Sdic2|NM_206793	sw|NM_206798	8/7	70/67	4	36
**Dme**	NM_079398_exon3::NM_057620_exon3	chr3L+17039679::chr2R-7162336	TpnC73F|NM_079398	TpnC47D|NM_057620	8/7	53/57	2	12
**Dme**	NM_130730_exon2::NM_166998_exon3	chrX+3833188::chrX+3841682	CG2941|NM_130730	CG32783|NM_166998	6/4	35/58	3	256
**Dme**	NM_167693_exon2::NM_079931_exon4	chrX-20087866::chrX-20069887	Sdic3|NM_167693	Sdic1|NM_079931	5/5	44/68	5	6
**Dme**	NM_167693_exon2::NM_057730_exon8	chrX-20087866::chrX-20101136	Sdic3|NM_167693	sw|NM_057730	5/5	44/68	5	6
**Dme**	NM_167693_exon2::NM_206793_exon4	chrX-20087866::chrX-20093262	Sdic3|NM_167693	Sdic2|NM_206793	5/5	44/68	5	5
**Dme**	NM_167693_exon2::NM_206793_exon4	chrX-20087866::chrX-20077703	Sdic3|NM_167693	Sdic2|NM_206793	5/5	44/68	5	5
**Dme**	NM_079931_exon2::NM_167693_exon4	chrX-20072303::chrX-20085420	Sdic1|NM_079931	Sdic3|NM_167693	5/5	44/68	5	4
**Dme**	NM_079931_exon2::NM_057730_exon8	chrX-20072303::chrX-20101136	Sdic1|NM_079931	sw|NM_057730	5/5	44/68	5	4

**Dme**	NM_079931_exon2::NM_206793_exon4	chrX-20072303::chrX-20093262	Sdic1|NM_079931	Sdic2|NM_206793	5/5	44/68	5	6
**Dme**	NM_079931_exon2::NM_206793_exon4	chrX-20072303::chrX-20077703	Sdic1|NM_079931	Sdic2|NM_206793	5/5	44/68	5	5
**Dme**	NM_057730_exon4::NM_167693_exon4	chrX-20105849::chrX-20085420	sw|NM_057730	Sdic3|NM_167693	5/5	44/68	5	4
**Dme**	NM_057730_exon4::NM_079931_exon4	chrX-20105849::chrX-20069887	sw|NM_057730	Sdic1|NM_079931	5/5	44/68	5	4
**Dme**	NM_057730_exon4::NM_206793_exon4	chrX-20105849::chrX-20093262	sw|NM_057730	Sdic2|NM_206793	5/5	44/68	5	5
**Dme**	NM_057730_exon4::NM_206793_exon4	chrX-20105849::chrX-20077703	sw|NM_057730	Sdic2|NM_206793	5/5	44/68	5	5
**Dme**	NM_206793_exon2::NM_167693_exon4	chrX-20095726::chrX-20085420	Sdic2|NM_206793	Sdic3|NM_167693	5/5	44/68	5	4
**Dme**	NM_206793_exon2::NM_079931_exon4	chrX-20095726::chrX-20069887	Sdic2|NM_206793	Sdic1|NM_079931	5/5	44/68	5	5
**Dme**	NM_206793_exon2::NM_057730_exon8	chrX-20095726::chrX-20101136	Sdic2|NM_206793	sw|NM_057730	5/5	44/68	5	3
**Dme**	NM_206793_exon2::NM_206793_exon4	chrX-20095726::chrX-20077703	Sdic2|NM_206793	Sdic2|NM_206793	5/5	44/68	5	5
**Dme**	NM_206793_exon2::NM_167693_exon4	chrX-20080167::chrX-20085420	Sdic2|NM_206793	Sdic3|NM_167693	5/5	44/68	5	4
**Dme**	NM_206793_exon2::NM_079931_exon4	chrX-20080167::chrX-20069887	Sdic2|NM_206793	Sdic1|NM_079931	5/5	44/68	5	5
**Dme**	NM_206793_exon2::NM_057730_exon8	chrX-20080167::chrX-20101136	Sdic2|NM_206793	sw|NM_057730	5/5	44/68	5	3
**Dme**	NM_206793_exon2::NM_206793_exon4	chrX-20080167::chrX-20093262	Sdic2|NM_206793	Sdic2|NM_206793	5/5	44/68	5	5
**Dme**	NM_142150_exon1::NM_078658_exon4	chr3R-10726675::chrX-17038749	RpS5b|NM_142150	RpS5a|NM_078658	4/4	66/66	1	17
**Dme**	NM_167590_exon3::NM_167590_exon6	chrX+17784706::chrX+17798831	CG32495|NM_167590	CG32495|NM_167590	3/3	58/69	3	8
**Dme**	NM_057276_exon1::NM_078583_exon2	chr2L+898941::chrX+12384693	Lsp1beta|NM_057276	Lsp1alpha|NM_078583	2/2	66/30	1	9
**Aae**	ACYPI001578-RA_exon4::ACYPI083436-RA_exon6	GL350277-420860::GL349863+177702	ACYPI001578|ACYPI001578-RA	ACYPI083436|ACYPI083436-RA	2/2	33/43	1	925
**Aae**	AAEL014108-RA_exon1::AAEL014255-RA_exon2	supercont1.1008-285166::supercont1.1050-260758	AAEL014108|AAEL014108-RA	AAEL014255|AAEL014255-RA	152/47	70/69	29	189
**Aae**	AAEL002358-RA_exon9::AAEL007083-RA_exon10	supercont1.54-3376813::supercont1.235+146572	AAEL002358|AAEL002358-RA	AAEL007083|AAEL007083-RA	138/31	70/70	19	803
**Aae**	AAEL007632-RA_exon1::AAEL010997-RA_exon2	supercont1.268+530222::supercont1.532-70244	AAEL007632|AAEL007632-RA	AAEL010997|AAEL010997-RA	15/10	69/58	7	21

**Aae**	AAEL005835-RA_exon3::AAEL014572-RA_exon5	supercont1.176+48330::supercont1.1171+209542	AAEL005835|AAEL005835-RA	AAEL014572|AAEL014572-RA	4/4	70/69	4	22
**Aae**	AAEL009652-RB_exon1::AAEL007384-RA_exon2	supercont1.415-49506::supercont1.252-1562316	AAEL009652|AAEL009652-RB	AAEL007384|AAEL007384-RA	4/4	38/66	2	14
**Aae**	AAEL005835-RA_exon4::AAEL014572-RA_exon4	supercont1.176+56536::supercont1.1171+208121	AAEL005835|AAEL005835-RA	AAEL014572|AAEL014572-RA	2/2	54/51	1	4
**Aae**	AAEL004926-RA_exon4::AAEL014216-RA_exon4	supercont1.135-46923::supercont1.1039+212650	AAEL004926|AAEL004926-RA	AAEL014216|AAEL014216-RA	2/2	58/44	1	5
**Aae**	AAEL014572-RA_exon4::AAEL005835-RA_exon4	supercont1.1171+208249::supercont1.176+56408	AAEL014572|AAEL014572-RA	AAEL005835|AAEL005835-RA	2/2	54/51	1	4
**Aae**	AAEL014572-RA_exon3::AAEL005835-RA_exon5	supercont1.1171+183549::supercont1.176+57991	AAEL014572|AAEL014572-RA	AAEL005835|AAEL005835-RA	2/2	70/40	2	6
**Aae**	AAEL014540-RA_exon1::AAEL010600-RA_exon2	supercont1.1156-111738::supercont1.489+864731	AAEL014540|AAEL014540-RA	AAEL010600|AAEL010600-RA	2/2	63/37	2	181
**Api**	ACYPI005232-RA_exon5::ACYPI002112-RA_exon8	GL350071+200193::GL349632+363940	ACYPI005232|ACYPI005232-RA	ACYPI002112|ACYPI002112-RA	61/27	44/39	10	164
**Api**	ACYPI009776-RA_exon1::ACYPI007903-RA_exon1	GL350578-168812::GL350578-147670	ACYPI009776|ACYPI009776-RA	ACYPI007903|ACYPI007903-RA	23/11	20/45	6	29
**Api**	ACYPI010087-RA_exon4::ACYPI003756-RA_exon6	GL350170-83966::GL349938+601539	ACYPI010087|ACYPI010087-RA	ACYPI003756|ACYPI003756-RA	8/3	31/35	1	588
**Api**	ACYPI008710-RA_exon4::ACYPI009234-RA_exon6	GL349662+538952::GL349911+226085	ACYPI008710|ACYPI008710-RA	ACYPI009234|ACYPI009234-RA	4/4	30/48	2	14
**Api**	ACYPI008207-RA_exon5::ACYPI009234-RA_exon6	GL349785-316055::GL349911+226085	ACYPI008207|ACYPI008207-RA	ACYPI009234|ACYPI009234-RA	4/4	30/48	2	15
**Api**	ACYPI000318-RA_exon3::ACYPI008622-RA_exon5	GL349797-288337::GL350241-33778	ACYPI000318|ACYPI000318-RA	ACYPI008622|ACYPI008622-RA	4/3	21/33	1	239
**Api**	ACYPI004235-RA_exon2::ACYPI003484-RA_exon4	GL350420+163611::GL350698-48308	ACYPI004235|ACYPI004235-RA	ACYPI003484|ACYPI003484-RA	3/3	46/40	1	609
**Api**	ACYPI003954-RA_exon9::ACYPI007694-RA_exon11	GL349832-538204::GL350655+15261	ACYPI003954|ACYPI003954-RA	ACYPI007694|ACYPI007694-RA	2/2	42/38	1	226
**Api**	ACYPI003484-RA_exon30::ACYPI004235-RA_exon26	GL350698-3038::GL350420+176135	ACYPI003484|ACYPI003484-RA	ACYPI004235|ACYPI004235-RA	3/3	37/26	1	634
**Api**	ACYPI003484-RA_exon25::ACYPI004235-RA_exon26	GL350698-27268::GL350420+176135	ACYPI003484|ACYPI003484-RA	ACYPI004235|ACYPI004235-RA	3/3	37/26	1	630
**Api**	ACYPI006288-RA_exon2::ACYPI006561-RA_exon4	GL349627-1601565::GL350609-25093	ACYPI006288|ACYPI006288-RA	ACYPI006561|ACYPI006561-RA	2/2	35/30	1	1264
**Api**	ACYPI40762-RA_exon1::ACYPI002463-RA_exon3	GL349901+64067::GL350249+82667	ACYPI40762|ACYPI40762-RA	ACYPI002463|ACYPI002463-RA	2/2	48/40	2	100
**Api**	ACYPI002463-RA_exon1::ACYPI40762-RA_exon3	GL350249+81996::GL349901+64734	ACYPI002463|ACYPI002463-RA	ACYPI40762|ACYPI40762-RA	2/2	48/40	1	79
**Api**	ACYPI083436-RA_exon4::ACYPI001578-RA_exon6	GL349863+175025::GL350277-417919	ACYPI083436|ACYPI083436-RA	ACYPI001578|ACYPI001578-RA	2/2	33/43	1	926

Supplementary Table 6. *Trans*-splicing events between paralog genes. Information of 60 *trans*-splicing events between paralog genes are listed. The homology of the donor genes and acceptor genes were determined by BLASTP program with 80% gene length coverage and 60% sequence similarity. Reads: all supporting reads and non-redundant supporting reads; Anchor: arm length of reads covering the junction site; Perfect: supporting reads with no mismatch; PE: paired-end supporting reads; Dme, *D. melanogaster*; Aae, *A. aegypti*; Api, *A. pisum*.

Supplementary References

1. Hordijk, W. & Gascuel, O. Improving the efficiency of SPR moves in phylogenetic tree search methods based on maximum likelihood. *Bioinformatics* **21**, 4338-4347 (2005).
2. Dorn, R., Reuter, G. & Loewendorf, A. Transgene analysis proves mRNA trans-splicing at the complex mod(mdg4) locus in Drosophila. *Proceedings of the National Academy of Sciences* **98** (2001).
3. Krauss, V. & Dorn, R. Evolution of the trans-splicing Drosophila locus mod(mdg4) in several species of Diptera and Lepidoptera. *Gene* **331**, 165-176 (2004).
4. Shao, W. *et al.* Alternative splicing and trans-splicing events revealed by analysis of the Bombyx mori transcriptome. *Rna* **18**, 1395-1407 (2012).
5. Lalitha., S. Primer Premier 5. *Biotech Software & Internet Report.* **1**, 270-272 (2000).
